# Quantum imaging of biological organisms through spatial and polarization entanglement

**DOI:** 10.1126/sciadv.adk1495

**Published:** 2024-03-08

**Authors:** Yide Zhang, Zhe He, Xin Tong, David C. Garrett, Rui Cao, Lihong V. Wang

**Affiliations:** Caltech Optical Imaging Laboratory, Andrew and Peggy Cherng Department of Medical Engineering, Department of Electrical Engineering, California Institute of Technology, Pasadena, CA 91125, USA.

## Abstract

Quantum imaging holds potential benefits over classical imaging but has faced challenges such as poor signal-to-noise ratios, low resolvable pixel counts, difficulty in imaging biological organisms, and inability to quantify full birefringence properties. Here, we introduce quantum imaging by coincidence from entanglement (ICE), using spatially and polarization-entangled photon pairs to overcome these challenges. With spatial entanglement, ICE offers higher signal-to-noise ratios, greater resolvable pixel counts, and the ability to image biological organisms. With polarization entanglement, ICE provides quantitative quantum birefringence imaging capability, where both the phase retardation and the principal refractive index axis angle of an object can be remotely and instantly quantified without changing the polarization states of the photons incident on the object. Furthermore, ICE enables 25 times greater suppression of stray light than classical imaging. ICE has the potential to pave the way for quantum imaging in diverse fields, such as life sciences and remote sensing.

## Teaser

Using spatially and polarization-entangled photon pairs, high-quality imaging of biological organisms can be achieved.

## INTRODUCTION

Since van Leeuwenhoek’s first microscope, optical imaging has been widely used to noninvasively investigate the structures and dynamics of various physical and biological systems (*1*, *2*). The key advantage of optical imaging is that the interaction of nonionizing light with molecules provides rich molecular information about biological samples. Aided by the convenience and compactness of optical systems, optical imaging has served as the workhorse for biological researchers and medical practitioners behind a wide variety of discoveries (*3*). In the past two decades, advanced optical imaging techniques have been developed to allow super-resolution (*1*, *4*) and high-speed (*5*, *6*) bioimaging. However, to achieve high-resolution and high-imaging speed, most optical imaging techniques require intense illumination that can disrupt or damage the biological processes under investigation (*2*). Low-intensity illumination may lead to a low signal-to-noise ratio (SNR) due to shot noise and stray light.

To overcome the limitations of existing optical imaging techniques that rely on classical light sources, quantum imaging approaches that use correlated, entangled, or squeezed photons have been developed (*7*–*13*). Compared with classical optical imaging, quantum imaging has the following advantages (*14*, *15*). First, the classical shot-noise limit can be surpassed, allowing for sub-shot-noise (SSN) imaging under low-intensity illumination (*12*, *13*, *16*–*23*). Second, stray light can be suppressed (*10*, *24*, *25*). Third, super-resolution imaging beyond the diffraction limit can be enabled (*8*, *26*–*32*). Empowered by these advantages, quantum imaging has been used to investigate biological specimens (*8*, *11*, *33*), which have complex structures and may be susceptible to photobleaching and thermal damage. Despite the advantages, quantum images of biological specimens reported to date still suffer low SNRs due to two main reasons. First, the conditions required to achieve SSN are stringent—e.g., demanding specimens with weak absorption or detectors with high quantum efficiency (*16*, *18*–*20*, *34*). Second, the accurate retrieval of coincidence rates necessitates a substantial number of measurements. Given the time constraints inherent in imaging processes, meeting this requirement for extensive measurements proves challenging, often leading to either inaccurate or noisy determinations of coincidence rates (*7*, *10*, *15*, *25*, *32*). Moreover, although megapixel widefield cameras exist (*35*–*37*), the resolvable pixel counts [i.e., the ratios of the field of view (FOV) to the spatial resolution area] of most quantum imaging approaches remain low, generally below 10,000 pixels (*7*–*11*). Besides, image tiling or stitching is not practical for the existing widefield methods because of the long acquisition time. Therefore, they are unsuitable for practical biological studies, which often demand systematic investigation of multiple parts in a biological system with an FOV across a whole organism. Last, while existing quantum imaging techniques have the capacity to measure transmittance (absorption) and birefringence phase retardation, the comprehensive quantification of birefringence properties through quantum imaging remains unachieved (*10*, *38*–*43*).

Here, we present imaging by coincidence from entanglement (ICE), a higher-SNR, greater-resolvable-pixel-count, and full-birefringence–quantified quantum imaging technique that generates high-quality images of biological specimens. Under low-intensity illumination, ICE uses an SSN algorithm that uses the covariance of the raw images to achieve a higher SNR than previous methods. Concurrently, ICE substantially increases the SNR over existing quantum imaging techniques by accommodating multiple spatial modes of the entangled photon pairs in each pixel, where a single spatial mode is constrained by the diffraction limit of the system (*44*, *45*). The spatial resolution of ICE is determined by both the signal and idler photons through a quantum effect named “entanglement pinhole.” In this effect, when an entangled photon pair is captured concurrently by two detectors, one detector functions nonclassically as a virtual pinhole on the object being imaged by the other detector. Further, ICE increases the resolvable pixel counts through raster scanning and is 25 times more resilient to stray light than classical imaging. Consequently, ICE enables quantum imaging of whole organ (mouse brain) slices and organisms (zebrafish) with an FOV of up to 7 mm × 4 mm and can be operated in the presence of ambient lighting, thus suitable for practical biological studies. Last, ICE exploits the polarization entanglement of the photon pairs for quantitative quantum birefringence imaging, where the full birefringence properties (including both the birefringence phase retardation and the principal refractive index axis angle) (*46*–*48*) of an object can be remotely and instantly quantified without changing the polarization states of the photons incident on the object. The quantum advantages of ICE, therefore, enable the observation of biological specimens under conditions that cannot be satisfied with classical imaging, as well as the remote sensing of full birefringence properties.

## RESULTS

### SSN quantum imaging using multimode entangled photons

**Fig. 1.**
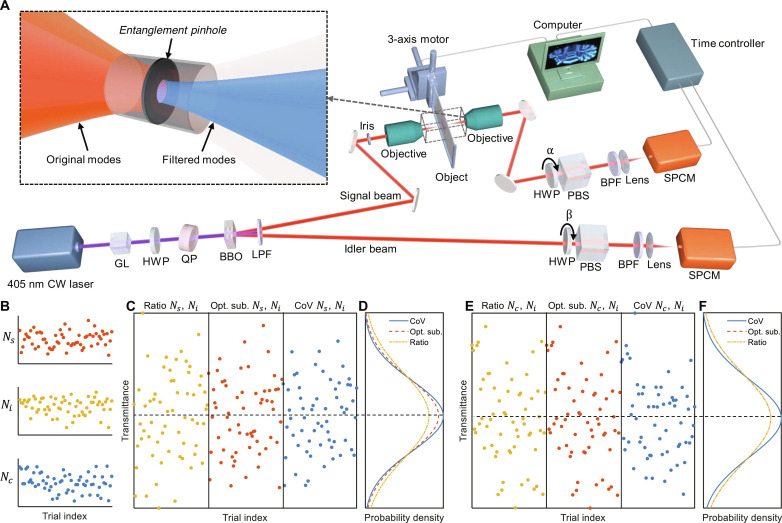
Experimental setup and SSN signal retrieval. (**A**) Setup schematics. CW, continuous wave; GL, Glan-Laser polarizer; HWP, half-wave plate; QP, quartz plate; BBO, β-barium borate crystals; LPF, long-pass filter; PBS, polarizing beam splitter; BPF, band-pass filter; SPCM, single-photon counting module. Inset, illustration of the entanglement pinhole. (**B**) Signal *N*_s_, idler *N_i_*, and coincidence *N*_c_ counts acquired from a series of trials. (**C**) Transmittance of the object experimentally measured using the ratio (eq. S2), optimized subtraction (eq. S4), and CoV (eq. S8) algorithms with *N*_s_ and *N*_i_. (**D**) Histograms of the transmittance measured in (C). (**E**) Transmittance of the object experimentally measured using the ratio, optimized subtraction, and CoV algorithms with *N*_c_ and *N*_i_. (**F**) Histograms of the transmittance measured in (E).

In ICE (Fig. 1A and Materials and Methods), we use two β-barium borate (BBO) nonlinear crystals with perpendicularly aligned optical axes to produce hyperentangled photon pairs, which are simultaneously entangled in spatial mode, polarization, and energy (*49*, *50*), through the type I spontaneous parametric down-conversion (SPDC) process. Most quantum imaging techniques reported to date evenly distribute the spatial modes of entangled photons across multipixel cameras (*10*, *16*, *25*, *51*, *52*), leading to a small number of spatial modes per pixel, a low coincidence rate, and, consequently, a low SNR in the image. In comparison, ICE increases the coincidence rate and SNR of quantum images by directly focusing the multimode SPDC beam onto the object, resulting in substantially more spatial modes in each pixel. ICE and existing quantum bioimaging techniques are quantitatively compared in Table 1. We record the signal (*N*_s_), idler (*N*_i_), and coincidence (*N*_c_) counts from the two single-photon counting modules (SPCMs) while raster scanning the object through the focused SPDC beam to image the transmittance of the object. Whereas *N*_s_ and *N*_c_ provide classical and quantum (ICE) images of the object, respectively, *N*_i_ can further improve the SNR of the images through SSN signal retrieval using our covariance-over-variance (CoV) algorithm (note S1 and figs. S1 to S3). Compared with state-of-the-art SSN methods such as ratio and optimized subtraction (*18*, *20*, *34*), our CoV algorithm achieves higher SNRs using either *N*_s_, *N*_i_ or *N*_c_, *N*_i_, as demonstrated through simulations (fig. S4) and experiments (Fig. 1, B to F).

**Table 1.** Comparison of ICE and existing quantum bioimaging modalities.

Work	Specimen	Specimen thickness	FOV	Resolvable pixel count	Image formation	Stray light resilience	SNR	$\hat{\text{SNR}}$ * (s^−0.5^)	Acquisition time per resolvable pixel (s)	Acquisition time per resolvable pixel for SNR = 10 (s)
Ref. (*7*)	Wasp wing section	<10 μm	4 × 4 mm^2^	2401	Widefield	N/A	6	7.2	0.7	1.9
Ref. (*8*)	NIH 3T3 cell microtubules	<1 μm	3 × 3 μm^2^	121	Scanning	N/A	12	9.5	1.6	1.1
Ref. (*9*)	Mouse heart section	<3 μm	1 × 1 mm^2^	784	Widefield	N/A	10	22.4	0.2	0.2
Ref. (*11*)	Yeast cell	<10 μm	10 × 10 μm^2^	2500	Scanning	N/A	5	11.2	0.2	0.8
Ref. (*10*)	Bird feather	<10 μm	2 × 1 mm^2^	968	Widefield	Yes	13	1.6	63	37.3
This work	Zebrafish/mouse brain	Up to 300 μm	Up to 7 × 4 mm^2^	Up to 258,432	Scanning	Yes	40	40	1	0.06

*Normalized SNR: $\hat{\text{SNR}}=\text{SNR}/\sqrt{\text{Acquisition time per resolvable pixel}}$.

**Fig. 2.**
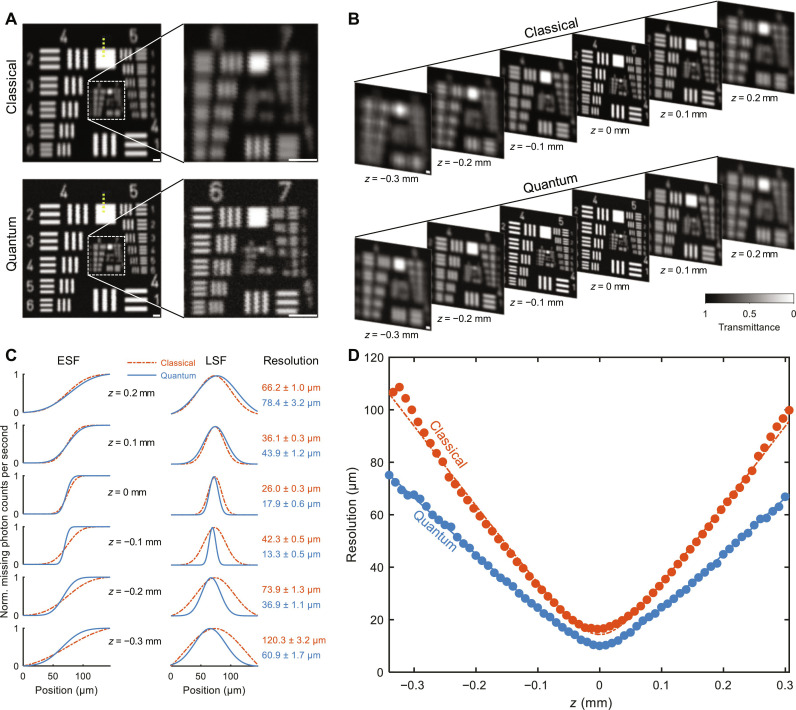
Effect of the entanglement pinhole on ICE. (**A** and **B**) Classical imaging and ICE of a USAF resolution target at focus (A) and at different *z* positions (B), where *z* = 0 mm denotes the focus of classical imaging. (**C**) ESFs, LSFs, and spatial resolutions measured at different *z* positions. The ESFs were fitted from the profiles along the yellow dotted lines in (A). The means and SEs of the resolution are shown on the right. (**D**) Resolution versus *z* for classical imaging and ICE. Dots represent experimental measurements. Solid and dash-dotted lines denote fits. Norm., normalized. Scale bars, 50 μm.

Despite the higher coincidence rate and SNR, acquiring images by raster scanning a multimode beam is generally undesired in classical imaging, as the multi-mode beam leads to a broad point spread function and, consequently, a poor spatial resolution. However, as shown in the inset of Fig. 1A, the spatially entangled photon pairs in ICE enable a quantum effect named “entangled pinhole,” where the detector in the idler arm functions as a pinhole on the object in the signal arm (note S2 and fig. S5). Because of the true coincidences from spatially entangled photons (note S3 and fig. S6), the entanglement pinhole improves the spatial resolution over classical imaging (fig. S7) while slightly increasing the depth of field (DOF). As shown in Fig. 2A, the classical image of a U.S. Air Force (USAF) resolution target can only resolve groups 4 and 5, whereas ICE can clearly resolve groups 6 and 7. Further, ICE maintains higher resolution over a long axial distance (Fig. 2B). To quantify the resolution and DOF experimentally, we acquired the edge spread functions (ESFs) of the images at different *z* positions. We then computed the line spread functions (LSFs) and their full width at half maximum (FWHM) to estimate the spatial resolutions (Materials and Methods). As shown in Fig. 2C, ICE has finer resolution than classical imaging from *z* = −0.3 mm to *z* = 0 mm. To calculate the DOF of the system, we repeated the same resolution analysis with a finer step size (10 μm) through approximately 700 μm along the *z* axis (Fig. 2D). To align the foci, the curve for ICE has been shifted to the right by 43 μm. By fitting the experimental data, the focal resolutions of classical imaging and ICE are determined to be 14.4 ± 0.6 μm and 10.4 ± 0.4 μm, respectively, demonstrating that ICE improves the resolution by 38% over classical imaging; the DOFs, on the other hand, are determined to be 92 ± 2 μm and 95 ± 2 μm for classical imaging and ICE, respectively. The 38% resolution enhancement achieved through virtual spatial filtering of the multimode SPDC beam with the entanglement pinhole, as described here and theoretically analyzed in note S2, is fundamentally distinct from the twofold super-resolution imaging at the Heisenberg limit demonstrated in (*10*, *32*). The latter relies on the principle that the equivalent wavelength of the entangled biphotons is half that of the SPDC photons.

**Fig. 3.**
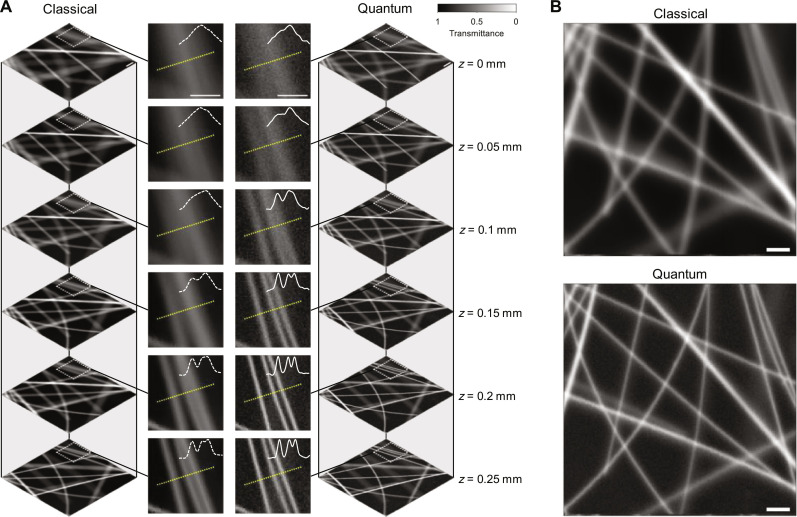
ICE of carbon fibers embedded in thick agarose. (**A**) Classical and ICE images of carbon fibers embedded in agarose at different *z* positions. Profiles along the yellow dotted lines are plotted in the close-ups to compare the spatial resolutions. (**B**) Average of the stacks in (A). Scale bars, 100 μm.

Compared with existing quantum imaging techniques that have been typically demonstrated with thin biological samples (e.g., <10 μm) (*7*–*11*), ICE provides a larger DOF, thus enabling the observation of thick objects. Here, we imaged 500-μm-thick agarose with randomly embedded carbon fibers of 6 μm diameter each. As shown in Fig. 3A, ICE can resolve the carbon fibers better than classical imaging throughout an axial range of 300 μm. The profiles along the yellow dashed lines demonstrate ICE’s ability to resolve three closely located fibers that cannot be clearly distinguished classically. ICE has imaged all targets in the object more clearly due to the higher spatial resolution and the slightly increased DOF over the classical counterpart. Specifically, comparing the averages of the three-dimensional (3D) stacks acquired classically and through ICE (Fig. 3B), one can see that, within a 3D volume of 1000 × 1000 × 300 μm^3^, the carbon fibers in the ICE stack are clearly better resolved than those in the classical stack.

### Quantum imaging of biological organisms in the presence of stray light

By raster scanning the object, ICE provides an FOV that can be conveniently extended. In contrast, while existing widefield quantum imaging techniques could potentially offer a similar large FOV by using methods such as image tiling or stitching, they necessitate the integration of high-precision translational stages, thereby extending the imaging duration substantially. In the low-illumination condition, widefield imaging requires longer integration time, which can present less optical throughput than that for ICE. This is because ICE directly measures coincidence using single-photon detection, while most widefield quantum imaging techniques use electron-multiplying charge-coupled devices, which do not have a high frame rate to measure the arrival time of a single photon, and therefore requires a large number of frames to retrieve the coincidence statistically (*7*, *10*, *32*). Single-photon avalanche diode (SPAD) array cameras are capable of facilitating direct coincidence measurements and have low dark count rates; however, they typically exhibit low quantum efficiency (*43*, *52*–*54*). Furthermore, while megapixel SPAD arrays are available (*35*, *36*), most of these arrays are equipped with a relatively low pixel count.

**Fig. 4.**
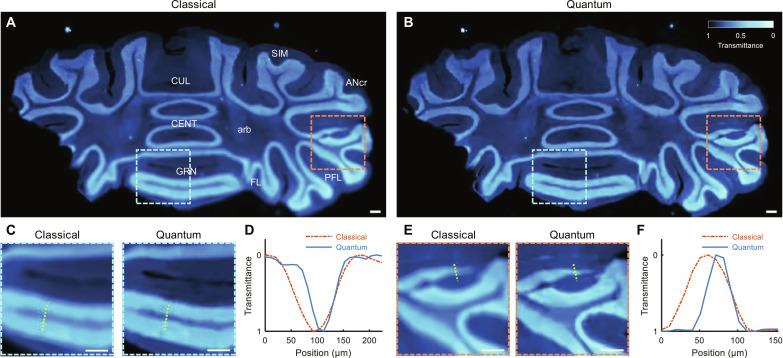
ICE of a mouse brain slice. (**A** and **B**) Classical (A) and ICE (B) images of a hematoxylin and eosin–stained mouse brain slice. ANcr, cerebellar hemisphere ansiform lobule crus; arb, arbor vitae; CENT, cerebellar vermis central lobule; CUL, cerebellar vermis culmen; FL, cerebellar hemisphere flocculus; GRN, gigantocellular reticular nucleus; PFL, cerebellar hemisphere paraflocculus; SIM, cerebellar hemisphere simple lobule. (**C**) Regions of interest (ROIs) denoted by the cyan rectangles in (A) and (B). (**D**) Profiles along the yellow dotted lines in (C). (**E**) ROIs denoted by the orange rectangles in (A) and (B). (**F**) Profiles along the yellow dotted lines in (E). Scale bars, 200 μm.

Here, we imaged a slice of a whole organ (the cerebellum of a mouse brain) with a 7 mm × 4 mm FOV, whose anatomical structures are annotated in Fig. 4A. The ICE image (Fig. 4B) outperforms the classical counterpart (Fig. 4A) with a higher resolution, as seen in the two regions of interest in Fig. 4 (C and E). Compared with the line profiles from the classical images (Fig. 4, D and F), the narrower trenches and peaks in the ICE profiles confirm an improved resolution across the large FOV.

**Fig. 5.**
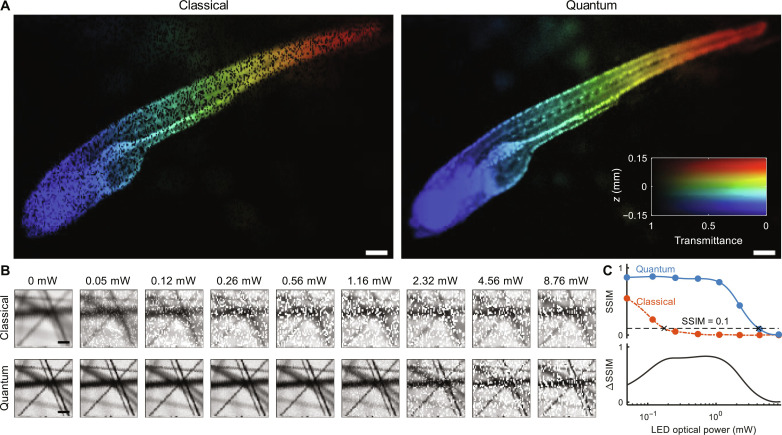
ICE in the presence of stray light. (**A**) Classical and ICE images of a whole zebrafish in the presence of stray light. The pseudo-colors encode the *z* positions of the sample. Scale bars, 200 μm. (**B**) Classical and ICE images of carbon fibers acquired at different stray light optical powers. Scale bars, 100 μm. (**C**) Top: SSIM calculated between the images in (B) and the ones without the stray light. Black dashed line, a threshold (SSIM = 0.1) used to quantify the robustness of ICE and classical imaging. Bottom: Difference between the SSIM curves for ICE and classical imaging.

In addition to the large FOV, ICE also demonstrates robust stray light resistance due to coincidence detection. To quantify ICE’s resilience to ambient lighting, a light-emitting diode (LED) was added to the system to introduce stray light (fig. S8). We acquired classical and ICE images of a biological organism, i.e., an agarose-embedded zebrafish, in a 3.5 mm × 2.3 mm FOV, while the LED was randomly turned on and off to simulate randomly fluctuating ambient light (Fig. 5). The zebrafish was positioned such that its torso was oblique to the imaging plane (fig. S9). As shown in Fig. 5A, while the classical imaging is severely degraded by the stray light, ICE is almost unaffected. We further quantify the robustness of ICE to stray light by acquiring a series of images of carbon fibers under different stray light intensities (Fig. 5B). Using the images acquired without the stray light as the ground truth, we calculated the structural similarity index measure (SSIM) of each image to quantify the degradation of the image quality due to stray light (Materials and Methods) (*55*). The SSIM is a figure of merit used to quantify the similarity between two images by evaluating changes in structural information, luminance, and contrast that align with human visual perception (*55*). It ranges from 0 to 1, where higher values indicate less degradation. The SSIM versus the stray light optical power is plotted in Fig. 5C. In accordance with the images in Fig. 5B, the classical images degrade quickly with an LED optical power above 0.1 mW, while ICE maintains a high SSIM even with an LED optical power above 1 mW. To simplify the comparison, we use an order-of-magnitude degradation (SSIM = 0.1) as a threshold to find the corresponding LED optical powers, found as 0.18 and 4.41 mW for the classical imaging and ICE, respectively. Therefore, ICE suppresses stray light 25 times more effectively than classical imaging. The advantage of ICE can also be seen in the difference between the two SSIM curves, i.e., ∆SSIM, shown in Fig. 5C. This advantage of ICE is attributed to coincidence detection, which is disturbed only by accidental coincidence counts. Despite its sufficient intensity to degrade a classical image, stray light acts as an uncorrelated source, causing negligible coincidence counts.

### Quantitative quantum birefringence imaging through polarization entanglement

Whereas most existing quantum imaging techniques rely on the spatial entanglement of SPDC photon pairs (*7*, *16*, *20*, *25*), quantum imaging modalities using polarization entanglement, such as the quantum holography (*10*), have been developed recently. The polarization entanglement of the SPDC photon pairs in our system can be characterized by Bell’s test (note S4 and fig. S10) (*56*, *57*). With an *S* value of 2.78 ± 0.01 > 2, our system shows a substantial violation of the Clauser-Horne-Shimony-Holt (CHSH) inequality (*58*), demonstrating strong polarization entanglement (*50*). By using hyperentangled photon pairs that are simultaneously entangled in spatial mode and polarization (*49*, *50*), ICE quantifies the full birefringence properties of an object without changing the polarization states of the photons incident on the object.

**Fig. 6.**
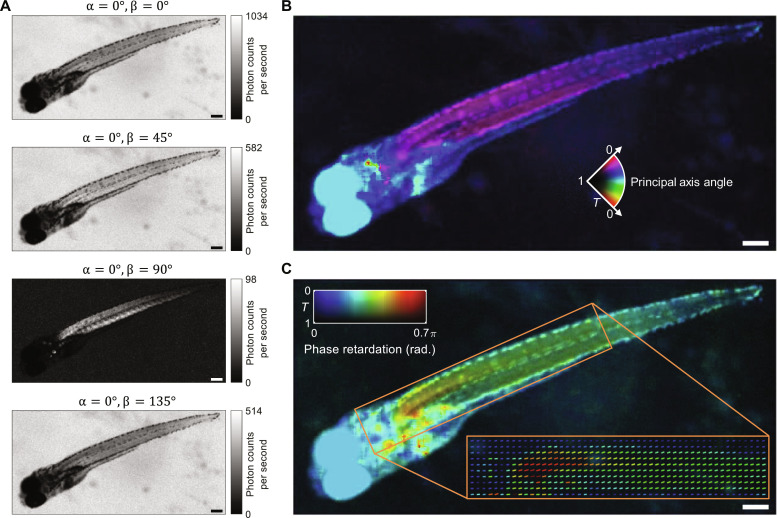
Quantitative quantum birefringence imaging of a whole zebrafish with ICE. (**A**) ICE images acquired with a polarizer of a constant angle α and a polarizer of a variable angle β. (**B**) Transmittance (*T*) and principal refractive index axis angle (pseudo-colors) calculated using the ICE images in (A). (**C**) Transmittance (*T*) and phase retardation between the two refractive index axes (lines and pseudo-colors) calculated using the ICE images in (A). Scale bars, 200 μm.

We evaluated the quantitative quantum birefringence imaging capability of ICE by imaging a biological organism—a whole zebrafish embedded in agarose. We kept the polarization of the signal photons incident on the object constant (α = 0°) while changing the polarization angles of the idler photons, which do not traverse the object, to four different angles (β = 0°, 45°, 90°, 135°). Whereas the four classical images exhibited little differences (fig. S11), the ICE images were substantially modulated by the birefringence properties of the zebrafish (Fig. 6A). Following the theory in note S5, the four ICE images could be used to calculate the transmittance, the angle of the principal refractive index (Fig. 6B), and the phase retardation between the two refractive index axes (Fig. 6C) of the zebrafish, providing full birefringence properties that are biologically relevant but have not been obtained with existing quantum imaging techniques (*10*, *38*–*43*). Furthermore, because of polarization entanglement, measuring the idler photon’s polarization state instantly determines the incident signal photons, thus allowing instant quantification of the object’s full birefringence properties, regardless of its distance. With the capability to remotely and instantly quantify the full birefringence properties (i.e., the Mueller matrix components) of a specimen by changing the polarization states of the photons that do not probe the object (i.e., “ghost birefringence quantification”), ICE can be used in remote sensing applications where the source is too far to be controlled in real time (fig. S12).

## DISCUSSION

Although imaging by coincidence can be achieved with a classical source (*59*), the SNR of the image will be 5.5 times lower compared to that of ICE under the same illumination intensity (note S6 and figs. S13 and S14), and the advantages enabled by spatial and polarization entanglement, such as SSN performance and ghost birefringence quantification, will be unavailable. We also note that, despite the similarity in using spatially entangled photon pairs and detecting coincidence for imaging, ICE fundamentally differs from ghost imaging (GI) (*60*–*62*) or correlation plenoptic imaging (CPI) (*63*, *64*) for the following reasons: (i) ICE generates a direct image of the object through raster scanning over a theoretically unlimited FOV, whereas GI and most CPI techniques provide an indirect, ghost image of the object by triggering a multipixel camera with a limited FOV; (ii) the signal arm of ICE contributes to spatial resolution, whereas the signal arms of GI and most CPI techniques do not; (iii) ICE images substantially more spatial modes per pixel than GI and CPI; and (iv) ICE exploits polarization entanglement in addition to the spatial entanglement used in GI and CPI (see note S7 and fig. S15 for detailed comparison).

Despite the advantages, ICE has the following limitations. First, limited by the low SPDC efficiency of the BBO crystal (*65*), the pixel dwell time is currently 1 s (i.e., a total acquisition time of 11.1 hours for an image with a pixel size of 200 × 200) for acquiring quantum images with an SNR of 40. If the SNR requirement is lower, the pixel dwell time will be shorter, e.g., 62.5 ms (i.e., a total acquisition time of 41.7 min for an image with a pixel size of 200 × 200) for an SNR of 10. It is also possible to use advanced analysis methods to extract information from images with even lower SNRs (e.g., SNR < 1) (*7*), which correspond to a pixel dwell time of less than 1 ms (i.e., a total acquisition time of 40 s for an image with a pixel size of 200 × 200). Second, because of the utilization of multimode SPDC beams, ICE has a lower spatial resolution compared to the Abbe limit of resolution (*1*, *2*), as evident in the comparison of direct classical (fig. S14B) and quantum coincidence (fig. S14C) images. These problems could be solved in the future by using more powerful quantum sources generated with, e.g., metalens arrays (*65*) or gated quantum dots in optical microcavities (*66*). A strong entangled photon source with high coincidence rates could substantially improve the imaging speed, and the SPDC beam could be filtered to a single spatial mode for diffraction-limited imaging while maintaining a sufficient SNR, which is extremely challenging to accomplish using existing quantum sources. Third, the entanglement pinhole is a virtual pinhole that filters SPDC modes in coincidence detection. In practice, all the SPDC photons in the signal arm still transmit through the object, which undergoes an illumination photon flux higher than the two-photon coincidence rate used for quantum imaging. Therefore, ICE is not a light-efficient imaging method. Nevertheless, the photon flux of all the SPDC photons on the object is less than 20,000 photons per second (fig. S14), which equals a total optical power of 4.9 × 10^−15^ W, an ultralow illumination that is safe for photosensitive biological specimens. Last, similar to other quantum imaging techniques using entangled photons, the imaging quality of ICE can deteriorate because of losses in the photodetection process. These losses can stem from inefficiencies within the optical path or the detector’s quantum efficiency (*20*, *67*). This challenge can be mitigated by using photodetectors characterized by high quantum efficiency.

To conclude, we have experimentally demonstrated ICE using hyperentangled photon pairs, achieving high-quality quantum bioimaging with higher SNR, greater resolvable pixel counts, and ghost birefringence quantification. As showcased using the thick biological organism (whole zebrafish) and the whole organ (mouse brain) slice with an FOV substantially larger than those of existing quantum images (fig. S16 and Table 1), these features allow for systematic observations in complex biological specimens. As shown in Table 1, ICE outperforms all other quantum bioimaging techniques with the highest normalized SNR and the shortest acquisition time per resolvable pixel for a given SNR. This advantage arises from ICE’s strategy of focusing a multimode SPDC beam directly onto the subject, thereby increasing spatial modes and coincidence rates per pixel. In contrast, other techniques distribute the spatial modes of SPDC beams across multipixel cameras, yielding fewer spatial modes per pixel and lower SNRs. Compared with existing quantum birefringence imaging techniques that provide either polarization-sensitive coincidence counts (*38*–*41*) or birefringence phase retardation images (*10*, *42*, *43*), ICE’s ghost birefringence quantification offers full birefringence properties, including not only the birefringence phase retardation but also the principal refractive index axis angle, both of which are important for biomedical imaging (fig. S17). Rather than competing with classical imaging techniques, ICE offers complementary benefits and additional opportunities such as SSN performance and ghost birefringence quantification, which cannot be accomplished with classical techniques. With these benefits and opportunities, ICE is expected to find more applications in life sciences where low illumination intensity [e.g., when probing delicate biological specimens like retinal rod cells (*68*)], ambient lighting, or precise measurements are required, and in remote sensing where the source cannot be controlled in real time.

## MATERIALS AND METHODS

### Experimental design

In our system (Fig. 1A), a paired set of BBO crystals [5 × 5 × 0.5 mm^3^ each, PABBO5050-405(I)-HA3, Newlight Photonics] was cut for type I SPDC at 405-nm wavelength. The two crystals were mounted back-to-back with one crystal rotated by 90° about the normal axis to the incidence surface. The pump was a 405-nm continuous wave laser (LM-405-PLR-40-4 K, Coherent) with an output power of 40 mW, which was chosen to avoid damage to the BBO crystals. A Glan-Laser polarizer (GL10-A, Thorlabs) and a half-wave plate (WPA03-H-405, Newlight Photonics) were used to adjust the pump laser beam to be linearly polarized at 45° relative to the vertical axis. A quartz plate (QAT25100-A, Newlight Photonics) tilted about its vertically oriented optical axis was used to precompensate for the phase difference between the horizontal and vertical polarization components of the SPDC photons. The pump laser beam then passed through the BBO crystals and generated a ring of SPDC photons with a half opening angle of 3°. A long-pass filter with a cut-on wavelength of 715 nm (LWPF1030-RG715, Newlight Photonics) was used to block the pump beam after the crystals. While the SPDC idler beam was directly sent to a half-wave plate (WPA03-H-810, Newlight Photonics) for polarization selection, the signal beam, whose size was adjusted by an iris (ID20, Thorlabs), was focused via an objective lens [LI-20×, 0.4 numerical aperture (NA); LI-10×, 0.25 NA; LI-4×, 0.1 NA; Newport] onto a microscope slide. The microscope slide was mounted on a three-axis motor (462-XYZ-M, each axis installed with an LTA-HS motorized actuator, Newport). The transmitted SPDC signal beam was collected by another objective lens of the same type and then sent to another half-wave plate (WPA03-H-810, Newlight Photonics) for polarization selection. The two half-wave plates were mounted on two motorized precision rotation mounts (PRM1Z8, Thorlabs), each followed by a polarizing beam splitter (PBS201, Thorlabs), an 810 ± 30–nm band-pass filter (NBF810-30, Newlight Photonics), a collection lens (LA1131, Thorlabs), and an SPCM (SPCM-AQRH-16, Excelitas Technologies). The two SPCMs were connected to a time controller (ID900-TCSPC-HR, ID Quantique) with a digital time resolution of 13 ps to measure both raw single-photon counts and coincidence counts. The time controller and the three-axis motor were synchronized and controlled by a computer. While motor-scanning the microscope slide holding the object, the raw single counts of the SPDC signal beam and the coincidence counts of the signal and idler beams were used to form the classical and ICE images of the object, respectively. The pixel dwell time was 1 s. The whole setup was covered by a light-shielding box.

### Characterization of polarization entanglement

The entanglement of the SPDC signal and idler photon pairs was evaluated using Bell’s test with the CHSH inequality (note S4) (*56*, *57*). Denoting the angles of the half-wave plates on the signal and idler paths as α and β, respectively, we recorded the coincidence counts *N*(α, β) at each step with an acquisition time of 1 s and a coincidence detection window of 8 ns. The correlation value was calculated by

$$E\left(\alpha ,\beta \right)=\frac{N\left(\alpha ,\beta \right)+N\left(\alpha +90{}^{\circ},\beta +90{}^{\circ}\right)-N\left(\alpha +90{}^{\circ},\beta \right)-N\left(\alpha ,\beta +90{}^{\circ}\right)}{N\left(\alpha ,\beta \right)+N\left(\alpha +90{}^{\circ},\beta +90{}^{\circ}\right)+N\left(\alpha +90{}^{\circ},\beta \right)+N\left(\alpha ,\beta +90{}^{\circ}\right)}$$

The CHSH inequality was then evaluated at the angle pairs α ∈ {0°, 45°} and β ∈ {22.5°, 67.5°} based on the value of *S* = ∣*E*(0°, 22.5°) − *E*(0°, 67.5°)∣ + ∣*E*(45°, 22.5°) + *E*(45°, 67.5°)∣. As shown in fig. S10, our system shows a strong violation of the CHSH inequity with *S* = 2.78 ± 0.01 > 2 estimated by calculating the mean and SE of *S* values measured from 10 rounds of Bell’s tests.

### Sample preparation

Four types of objects have been imaged. The wild-type zebrafish was fixed by 4% paraformaldehyde (PFA) solution 5 days after fertilization. After fixation, the zebrafish was washed three to four times using phosphate-buffered saline in a fume hood before agarose embedding. The agarose-embedded zebrafish was mounted onto a glass slide and sealed with a coverslip to prevent dehydration during the experiment. To prepare the brain slice, a brain was obtained from a Swiss Webster mouse (Hsd: ND4, Harlan Laboratories) and fixed in 3.7% PFA solution at room temperature for 24 hours. After paraffin embedding, coronal sections (10 μm thick) of the brain were cut. Standard hematoxylin and eosin staining was performed on the sections, which were examined using a bright-field microscope (NanoZoomer, Hamamatsu) with a 20 × 0.67 NA objective lens. All animal procedures were approved by the Institutional Animal Care and Use Committee of California Institute of Technology. We used a 2″ × 2″ positive 1951 USAF resolution target (58–198, Edmund Optics) to quantify the spatial resolution and DOF of our system. To prepare the thick object, carbon fibers with a diameter of 6 μm were randomly embedded in a 4% agarose block (A-204-25, GoldBio) in 3D. A 500-μm-thick section was created from the agarose block using a vibratome (VT1200S, Leica). Next, the section was placed onto a standard microscope glass slide and fixed by applying cyanoacrylate glue around the edge. A coverglass was put on top of the sample and sealed using epoxy glue to prevent dehydration of the agarose.

### Data acquisition and processing

A custom-written LabVIEW (National Instruments) program was used to synchronize the raster scanning of the three-axis motor with the data acquisition of the time controller and acquire the raw singles and coincidence counts of the two SPCMs. When acquiring 2D imaging data, the LabVIEW program raster scanned the *x*- and *y*-axis motors and converted the raw single counts of the signal channel and coincidence counts into classical and ICE images, respectively. The images were displayed on screen and saved to the computer in tag image file format (TIF). For imaging thick objects, multiple 2D images each captured at a *z* position were combined to form a 3D stack. The TIF files were imported into MATLAB (MathWorks) and processed with custom-written scripts. Depending on the objects being imaged, the images were rotated, cropped, or inverted before being used to extract line profiles or ESFs for estimating resolution and DOF. In addition, to compensate for the low contrast between the brain structure and the background, the brain slice images were denoised by block-matching and 3D filtering (*69*) followed by a variance-stabilizing transformation (*70*).

### Measurements of resolution and DOF

To measure the spatial resolution of our system, the profile of a line along *x* perpendicular to an edge in the USAF resolution target (e.g., the yellow dashed line in Fig. 2A) was extracted and fitted to an ESF centered at *x*_0_, i.e., ESF(*x*) = *a* erf ((*x* − *x*_0_)/*w*) + *b*, where *a* and *b* are coefficients, and *w* is the radius of the beam. A Gaussian LSF was obtained by taking the derivative of the ESF, i.e., $\text{LSF}\left(x\right)=d\text{ESF}\left(x\right)/\mathrm{dx}=2a\text{exp}\left(-{\left(x-x_{0}\right)}^{2}/w^{2}\right)/\left(w\sqrt{\pi }\right)$ . The resolution was estimated to be the FWHM of the LSF, i.e., $ℛ=2\sqrt{\text{ln}2}w$ . The mean value of the resolution was estimated to be $2\sqrt{\text{ln}2}$ times the fitted *w*, and the SE was calculated to be $\sqrt{\text{ln}2}/1.96$ times the 95% confidence interval of the fitted *w*. To measure the DOF of our system, resolution, *ℛ*, was estimated at each *z* position (e.g., Fig. 2D). The curves were fitted for *z_R_* to a hyperbolic function, i.e., $ℛ\left(z\right)={ℛ}_{0}\sqrt{1+{\left(z-z_{0}\right)}^{2}/z_{R}^{2}}$ , where *ℛ*_0_ is the focal resolution and *z_R_* is the Rayleigh length. The mean DOF was estimated to be 2*z_R_*, and the SE was estimated to be 1/1.96 times the 95% confidence interval of the fitted *z_R_*.

### Imaging with stray light

A white LED (MNWHL4, Thorlabs) powered by an LED driver (DC2200, Thorlabs) was used to randomly generate stray light during imaging, as shown in fig. S8. The LED driver was externally triggered by an analog output device (PCI-6711, National Instruments) installed on the computer. While raster scanning the object prepared on the microscope slide, at each pixel, the LabVIEW program generated a random number uniformly distributed between 0 and 1 to determine whether to trigger the white LED to output stray light. If the random number was less than 0.2, the LED was triggered to generate stray light; otherwise, no stray light would be generated. Therefore, approximately 20% of the pixels would be disrupted by stray light. To evaluate how robust the classical imaging and ICE were against stray light, we acquired images under different stray light optical powers. We calculated the SSIM between each image and the ground truth at zero stray light by $\text{SSIM}=4\mu_{1}\mu_{2}\sigma_{12}/((\mu_{1}^{2}+\mu_{2}^{2})(\sigma_{1}^{2}+\sigma_{2}^{2}))$ , where μ*_i_* and $\sigma_{i}^{2}$ (*i* = 1 or 2) are the average and variance of each image, respectively, and *σ*_12_ is the covariance of the two images (*55*). We used SSIM as the figure of merit because it aligns well with human visual perception, provides robust and accurate assessments, and is versatile across a wide range of applications and different reference availability situations (*55*).

## Supplementary Materials

### This PDF file includes:

Notes S1 to S7 Figs. S1 to S17 References
