# Supplementary material for: Quantum imaging of biological organisms through spatial and polarization entanglement
Source: Sci Adv. 2024 Mar 8;10(10):eadk1495. doi: 10.1126/sciadv.adk1495 (PMC10923495; doi:10.1126/sciadv.adk1495)
Supplement: Supplementary file 1 — Notes S1 to S7 Figs. S1 to S17 References [file sciadv.adk1495_sm.pdf]

Science Advances
AAAS

Supplementary Materials for

Quantum imaging of biological organisms through spatial and polarization entanglement

Yide Zhang *et al.*

Corresponding author: Lihong V. Wang, lvw@caltech.edu

Sci. Adv. **10**, eadk1495 (2024)
DOI: 10.1126/sciadv.adk1495

This PDF file includes:

Notes S1 to S7
Figs. S1 to S17
References

Note S1 Sub-shot-noise signal retrieval in ICE

Each round of ICE acquisition generates three images: the signal image $N_s(\boldsymbol{r})$, the idler image $N_i(\boldsymbol{r})$, and the coincidence image $N_c(\boldsymbol{r})$. $N_s(\boldsymbol{r})$ and $N_i(\boldsymbol{r})$ contain photon counts from both SPDC photon pairs (whose averaged value is denoted as μ_{SPDC}) and stray light (whose averaged value is denoted as μ_{stray}). For simplicity, we assume the signal and idler detectors have the same background light intensity and detection efficiency, denoted as η.

The imaging of an object here measures its transmittance $T(\boldsymbol{r})$. Although the following derivation applies to both N_s and N_c, we use N_s as an example. Classically, the transmittance is estimated as

$$T_0(\boldsymbol{r}) = \frac{N_s(\boldsymbol{r})}{N_s^0(\boldsymbol{r})}, \tag{S1}$$

where N_s^0 denotes the signal image when the object is absent. In ICE, we estimate N_s^0 using $\langle N_s^{\mathrm{b}}(\boldsymbol{r})\rangle_{\boldsymbol{r}}$, where N_s^{b} denotes a background region of the N_s image outside the target, and $\langle\dots\rangle_{\boldsymbol{r}}$ denotes averaging over spatial locations.

By using the correlation between the SPDC photon pairs, two types of sub-shot-noise (SSN) algorithms have been adopted to enhance the SNR of the transmittance measurements. The first type relies on the ratio of the two images, where the object's transmittance is estimated as (*34*, *71*)

$$T_1(\boldsymbol{r}) = \frac{N_s(\boldsymbol{r})}{N_i(\boldsymbol{r})} \cdot \frac{\langle N_i(\boldsymbol{r})\rangle_{\boldsymbol{r}}}{\langle N_s^{\mathrm{b}}(\boldsymbol{r})\rangle_{\boldsymbol{r}}}. \tag{S2}$$

The second type of SSN algorithms, termed optimized subtraction, suppresses the noise in N_s by subtracting the variation of N_i (*18*, *72*):

$$N_s^{\mathrm{SSN}}(\boldsymbol{r}) = N_s(\boldsymbol{r}) - k(\boldsymbol{r})\Delta N_i(\boldsymbol{r}), \tag{S3}$$

where $k(\boldsymbol{r})$ is the unknown spatially varying multiplier, and $\Delta N_i(\boldsymbol{r}) = N_i(\boldsymbol{r}) - \langle N_i(\boldsymbol{r})\rangle_{\boldsymbol{r}}$. The ideal $k(\boldsymbol{r})$ is proportional to the transmittance $T(\boldsymbol{r})$, the ground truth of which is unknown. To estimate the $k(\boldsymbol{r})$, one may use the approximated transmittance $\hat{T}(\boldsymbol{r})$. For example, $\hat{T}(\boldsymbol{r})$ can be acquired using T_0 as in Eq. (S1) or as in Ref. (*18*). The object's transmittance estimated using the second type of SSN algorithms is given by

$$T_2(\boldsymbol{r}) = \frac{N_s(\boldsymbol{r})}{\langle N_s^{\mathrm{b}}(\boldsymbol{r})\rangle_{\boldsymbol{r}}} - \hat{T}(\boldsymbol{r})\eta\left(\frac{\mu_{\mathrm{SPDC}}}{\mu_{\mathrm{stray}} + \mu_{\mathrm{SPDC}}}\right)^2 \frac{\Delta N_i(\boldsymbol{r})}{\langle N_s^{\mathrm{b}}(\boldsymbol{r})\rangle_{\boldsymbol{r}}}. \tag{S4}$$

Both Eqs. (S2) and (S4) achieve higher SNR than Eq. (S1), demonstrating the quantum advantage. However, these methods require either prior knowledge of $T(\boldsymbol{r})$ or assumptions on the photon distribution and minimal stray light intensity. Here, inspired by the two algorithms, we introduce the covariance-over-variance (CoV) algorithm to further improve the SSN performance with fewer assumptions.

The workflow of the CoV algorithm is shown in Fig. S1. We acquire the time-lapsed image stack of $N_s(\boldsymbol{r},t)$, $N_i(\boldsymbol{r},t)$, and $N_c(\boldsymbol{r},t)$. Following the basic framework of the optimized subtraction (i.e., Eq. (S3)), instead of estimating $k(\boldsymbol{r})$ with approximated $T(\boldsymbol{r})$, we derive the optimal $k(\boldsymbol{r})$ by minimizing the variance of $N_s^{\mathrm{SSN}}(\boldsymbol{r},t)$:

$$\mathrm{Var}_t[N_s^{\mathrm{SSN}}(\boldsymbol{r},t)] = \mathrm{Var}_t[N_s(\boldsymbol{r},t)] + k^2(\boldsymbol{r})\mathrm{Var}_t[N_i(\boldsymbol{r},t)] - 2k(\boldsymbol{r})\mathrm{Cov}_t[N_s(\boldsymbol{r},t), N_i(\boldsymbol{r},t)], \tag{S5}$$

where Var_t and Cov_t denote the variance and covariance along the time sequence, respectively.

To minimize $\mathrm{Var}_t[N_s^{\mathrm{SSN}}(\boldsymbol{r},t)]$ with regard to $k(\boldsymbol{r})$:

$$\frac{\partial \mathrm{Var}_t[N_s^{\mathrm{SSN}}(\boldsymbol{r},t)]}{\partial k(\boldsymbol{r})} = 2k(\boldsymbol{r})\mathrm{Var}_t[N_i(\boldsymbol{r},t)] - 2\mathrm{Cov}_t[N_s(\boldsymbol{r},t), N_i(\boldsymbol{r},t)] = 0. \tag{S6}$$

The optimized $k^*(\boldsymbol{r})$ is thus given by

$$k^*(\boldsymbol{r}) = \frac{\mathrm{Cov}_t[N_s(\boldsymbol{r},t), N_i(\boldsymbol{r},t)]}{\mathrm{Var}_t[N_i(\boldsymbol{r},t)]}. \tag{S7}$$

Since $\langle N_s^{\mathrm{SSN}}(\boldsymbol{r})\rangle_{\boldsymbol{r}} = \langle N_s(\boldsymbol{r})\rangle_{\boldsymbol{r}}$ according to Eq. (S3), combining Eqs. (S1), (S3), and (S7) completes the CoV algorithm:

$$T_3(\boldsymbol{r}) = \frac{N_s(\boldsymbol{r})}{\langle N_s^{\mathrm{b}}(\boldsymbol{r})\rangle_{\boldsymbol{r}}} - \frac{\mathrm{Cov}_t[N_s(\boldsymbol{r},t), N_i(\boldsymbol{r},t)]}{\mathrm{Var}_t[N_i(\boldsymbol{r},t)]} \cdot \frac{\Delta N_i(\boldsymbol{r})}{\langle N_s^{\mathrm{b}}(\boldsymbol{r})\rangle_{\boldsymbol{r}}}. \tag{S8}$$

From Eqs. (S5) and (S7), we can derive the minimized variance as

$$\mathrm{Var}_t[N_s^{\mathrm{SSN}}(\boldsymbol{r},t)] = \mathrm{Var}_t[N_s(\boldsymbol{r},t)] - \frac{\mathrm{Cov}_t^2[N_s(\boldsymbol{r},t), N_i(\boldsymbol{r},t)]}{\mathrm{Var}_t[N_i(\boldsymbol{r},t)]} = \mathrm{Var}_t[N_s(\boldsymbol{r},t)]\left(1-\rho_{N_s,N_i}^2\right), \tag{S9}$$

where ρ_{N_s,N_i} is the Pearson's correlation coefficient between N_s and N_i along the time sequence. Note that the variance of the classical algorithm given by Eq. (S1) is $\mathrm{Var}_t[N_s(\boldsymbol{r},t)]$. Since $\rho_{N_s,N_i}^2 \geq 0$, the CoV algorithm guarantees SNR enhancement.

It is worth noting that the CoV algorithm requires repeated measurements over time. With a single-frame acquisition, we propose a similar algorithm to estimate $k(\boldsymbol{r})$ based on spatial repetitions, named as the s-CoV algorithm.

The workflow of the s-CoV algorithm is shown in Fig. S2. From the single-frame image $N_s(\boldsymbol{r})$ (or $N_c(\boldsymbol{r})$), we calculate the histogram and divide the pixel values into L bins. The selection of L depends on the experimental configuration and can be optimized through iteration. For the l-th bin ($l = 1,2,\dots,L$), we select the pixels from the image whose values fall into this bin and form the image subset $N_s^l(\boldsymbol{r})$. The binary mask $M^l(\boldsymbol{r})$ used for segmentation (i.e., $N_s^l(\boldsymbol{r}) = M^l(\boldsymbol{r}) \cdot N_s(\boldsymbol{r})$) is then applied to $N_i(\boldsymbol{r})$ to get the image subset $N_i^l(\boldsymbol{r})$. Following Eq. (S7), we can estimate the subset of $k^*(\boldsymbol{r})$ (denoted as $k^{n,*}$) as

$$k^{l,*} = \frac{\mathrm{Cov}_{\boldsymbol{r}}\left[N_s^l(\boldsymbol{r}), N_i^l(\boldsymbol{r})\right]}{\mathrm{Var}_{\boldsymbol{r}}[N_i^l(\boldsymbol{r})]}, \tag{S10}$$

where $\mathrm{Var}_{\boldsymbol{r}}$ and $\mathrm{Cov}_{\boldsymbol{r}}$ denote the variance and covariance along spatial locations, respectively. The same procedure is repeated for all l, and the resulting $k^*(\boldsymbol{r})$ is the summation of all $M^l(\boldsymbol{r})$ modified by $k^{l,*}$:

$$k^*(\boldsymbol{r}) = \sum_l k^{l,*} M^l(\boldsymbol{r}) = \sum_l \frac{\mathrm{Cov}_{\boldsymbol{r}}\left[N_s^l(\boldsymbol{r}), N_i^l(\boldsymbol{r})\right]}{\mathrm{Var}_{\boldsymbol{r}}[N_i^l(\boldsymbol{r})]} M^l(\boldsymbol{r}). \tag{S11}$$

Combining Eqs. (S1), (S3), and (S11) completes the s-CoV algorithm:

$$T_4(\boldsymbol{r}) = \frac{N_s(\boldsymbol{r})}{\langle N_s^{\mathrm{b}}(\boldsymbol{r})\rangle_{\boldsymbol{r}}} - \left[\sum_l \frac{\mathrm{Cov}_{\boldsymbol{r}}\left[N_s^l(\boldsymbol{r}), N_i^l(\boldsymbol{r})\right]}{\mathrm{Var}_{\boldsymbol{r}}[N_i^l(\boldsymbol{r})]} M^l(\boldsymbol{r})\right] \cdot \frac{\Delta N_i(\boldsymbol{r})}{\langle N_s^{\mathrm{b}}(\boldsymbol{r})\rangle_{\boldsymbol{r}}}. \tag{S12}$$

A comparison of the workflows of the ratio, optimized subtraction, and CoV algorithms is shown

in Fig. S3. To compare the performances of the three algorithms, we simulate the 1D case where the object is placed in the signal arm with constant $T(x) = 0.5$. Each detector in the signal and idler arms performs Bernoulli trials with probability η to select the SPDC photons (following a Poisson distribution with μ_{SPDC}). The stray light photons in each detector follow two independent Poisson distributions with $\eta\mu_{\mathrm{stray}}$. The schematics are shown in Fig. S4A. We simulate two scenarios with (1) a fixed stray light-SPDC light ratio ($\mu_{\mathrm{stray}}/\mu_{\mathrm{SPDC}} = 1$) and a varying detector efficiency η and (2) a fixed $\eta = 0.7$ and a varying $\mu_{\mathrm{stray}}/\mu_{\mathrm{SPDC}}$. Fig. S4, B and C, show the SNR enhancement using N_s and N_i images with Eqs. (S2), (S4), and (S8) compared to Eq. ($S1$). Fig. S4, D and E, show similar results using N_c and N_i images. We also experimentally compared the SNR performances of the three algorithms by acquiring 60 trials of N_s, N_i, and N_c and implemented the ratio (Eq. (S2)), optimized subtraction (Eq. ($S4$)), and CoV (Eq. ($S8$)) algorithms with them (Fig. 1B). Fig. 1, C and D, show that the transmittance measured using N_s and N_i with the CoV algorithm has the highest SNR compared to the ratio and optimized subtraction algorithms. Fig. 1, E and F, show similar results using N_c and N_i. In all cases, the CoV algorithm outperforms the others consistently.

Note S2 Entanglement pinhole in ICE

In the simplified schematic of the imaging system shown in Fig. S5, $\boldsymbol{r}_{0,s}$, $\boldsymbol{r}_{1,s}$, and $\boldsymbol{r}_{2,s}$ represent the coordinates of the BBO, the object, and the two detectors D_s for signal photons, respectively. $\boldsymbol{r}_{0,i}$ and $\boldsymbol{r}_{2,i}$ represent the transverse coordinates of the BBO and the detector D_i for idler photons, respectively. $\boldsymbol{k}_{0,s}$ and $\boldsymbol{k}_{0,i}$ represent the wavevectors of the entangled signal and idler photons emitted from the BBO, respectively. $\boldsymbol{k}_{1,s}$ denotes the wavevector of the signal photon after the first objective. $\boldsymbol{k}'_{1,s}$ denotes the wavevector of the signal photon emitted from the object. $\boldsymbol{k}_{2,s}$ denotes the wavevector of the signal photon on the detector D_s. All wavevectors have the same constant magnitude k. Here, the subscripts *s* and *i* denote signal and idler, respectively.

The phase-matching condition constrains that the transverse components of $\boldsymbol{k}_{0,s}$ and $\boldsymbol{k}_{0,i}$ are opposite while their axial components are identical. If $\boldsymbol{r_p}$ and $\boldsymbol{k_p}$ represent the position and wavevector of the pump light, the state denotes a spatially entangled state with $(\boldsymbol{r}_{0,s} + \boldsymbol{r}_{0,i})/2 = \boldsymbol{r_p}$ and $\boldsymbol{k}_{0,s} + \boldsymbol{k}_{0,i} = \boldsymbol{k_p}$. Because of the latter constraint, we choose to sum over $\boldsymbol{k}_{0,s}$ only in the following derivation. In the signal arm, $\hat{O}^s$ and $\hat{O}^{s'}$ denote the operators for the objective and lenses before and after the object plane, respectively. In the idler arm, $\hat{O}^i$ denotes the operators for the lens in front of D_i. For simplicity, we set the image magnification ratios of both channels to unity. $\hat{E}_s^{(-)}$ and $\hat{E}_i^{(-)}$ are the Hermitian conjugates of the electric fields $\hat{E}_s^{(+)}$ and $\hat{E}_i^{(+)}$, respectively. The electric fields are derived by propagation from the source to the detectors as follows (*73*):

$$\hat{E}_s^{(+)}(\boldsymbol{r}_{2,s}, \boldsymbol{r}_{0,s}; \boldsymbol{r}_{1,s}) = |0_{\boldsymbol{r}_{2,s}}\rangle\langle 1_{\boldsymbol{r}_{2,s}}|\hat{O}^{s'}|1_{\boldsymbol{r}_{1,s}}\rangle t_0 \langle 1_{\boldsymbol{r}_{1,s}}|\hat{O}^s E_0(\boldsymbol{r}_{0,s})\hat{e}, \quad (S13)$$

$$\hat{E}_i^{(+)}(\boldsymbol{r}_{2,i}, \boldsymbol{r}_{0,i}) = |0_{\boldsymbol{r}_{2,i}}\rangle\langle 1_{\boldsymbol{r}_{2,i}}|\hat{O}^i E_0(\boldsymbol{r}_{0,i})\hat{e}. \quad (S14)$$

Here, E_0 is the amplitude of the electric field; $\hat{e}$ is the polarization unit vector; t_0 is the amplitude transmission coefficient of the object. The projectors $|0_{\boldsymbol{r}_{2,s}}\rangle\langle 1_{\boldsymbol{r}_{2,s}}|$ and $|0_{\boldsymbol{r}_{2,i}}\rangle\langle 1_{\boldsymbol{r}_{2,i}}|$ account for measurements at $\boldsymbol{r}_{2,s}$ and $\boldsymbol{r}_{2,i}$, respectively. The type-I SPDC crystal guarantees the same polarization $\hat{e}$ for the signal and idler photons.

The wavefunction $|\xi\rangle$ for each entangled photon pair is defined as

$$|\xi\rangle = \sum_{\boldsymbol{k}_{0,s}} e^{-j\boldsymbol{k}_{0,s}\cdot\boldsymbol{r}_{0,s}} e^{-j\boldsymbol{k}_{0,i}\cdot\boldsymbol{r}_{0,i}} \left|1_{\boldsymbol{k}_{0,s}}, 1_{\boldsymbol{k}_{0,i}}\right\rangle. \tag{S15}$$

Next, we compute the field correlation using Eqs. (S13)–(S15):

$$F = \left\langle 0_{\boldsymbol{r}_{2,s}}, 0_{\boldsymbol{r}_{2,i}}\right|\hat{E}_i^{(+)}(\boldsymbol{r}_{2,i}, \boldsymbol{r}_{0,i})\hat{E}_s^{(+)}(\boldsymbol{r}_{2,s}, \boldsymbol{r}_{0,s}; \boldsymbol{r}_{1,s})|\xi\rangle$$
$$= E_0^2 \sum_{\substack{\boldsymbol{k}_{0,s}, \boldsymbol{k}_{1,s}, \boldsymbol{k}'_{1,s}, \boldsymbol{k}_{2,s} \\ \boldsymbol{k}_{2,i}}} t_0 h_k(\boldsymbol{r}_{2,s}, \boldsymbol{k}_{2,s}; \boldsymbol{r}_{1,s}, \boldsymbol{k}'_{1,s}) h_k(\boldsymbol{r}_{0,s}, \boldsymbol{k}_{0,s}; \boldsymbol{r}_{1,s}, \boldsymbol{k}_{1,s}) h_k(\boldsymbol{r}_{2,i}, \boldsymbol{k}_{2,i}, \boldsymbol{r}_{0,i}, \boldsymbol{k}_{0,i}). \tag{S16}$$

Here, $h_k(\boldsymbol{r}_{0,s}, \boldsymbol{k}_{0,s}; \boldsymbol{r}_{1,s}, \boldsymbol{k}_{1,s})$ represents the photon propagation from $\boldsymbol{r}_{0,s}$ to $\boldsymbol{r}_{1,s}$ through the objective $\hat{O}^s$ with the vectors $\boldsymbol{k}_{0,s}$ and $\boldsymbol{k}_{1,s}$. Other h_k functions are interpreted and derived similarly. As an example, h_k is derived as follows:

$$\left\langle 1_{\boldsymbol{r}_{1,s}}\middle|\hat{O}^s\middle|1_{\boldsymbol{r}_{0,s}}\right\rangle = \sum_{\boldsymbol{k}_{0,s}, \boldsymbol{k}_{1,s}} \left\langle 1_{\boldsymbol{r}_{1,s}}\middle|\middle|1_{\boldsymbol{k}_{1,s}}\right\rangle\left\langle 1_{\boldsymbol{k}_{1,s}}\middle|\hat{O}^s\middle|1_{\boldsymbol{k}_{0,s}}\right\rangle\left\langle 1_{\boldsymbol{k}_{0,s}}\middle|\middle|1_{\boldsymbol{r}_{0,s}}\right\rangle$$
$$= \sum_{\boldsymbol{k}_{0,s}, \boldsymbol{k}_{1,s}} h_k(\boldsymbol{r}_{0,s}, \boldsymbol{k}_{0,s}; \boldsymbol{r}_{1,s}, \boldsymbol{k}_{1,s}). \tag{$S17$}$$

The h_k functions are related to the phase shifts for the signal and idler arms:

$$h_k(\boldsymbol{r}_{0,s}, \boldsymbol{k}_{0,s}; \boldsymbol{r}_{1,s}, \boldsymbol{k}_{1,s}) = e^{i\phi_{s,10}}, \tag{S18}$$
$$h_k(\boldsymbol{r}_{2,s}, \boldsymbol{k}_{2,s}; \boldsymbol{r}_{1,s}, \boldsymbol{k}'_{1,s}) = e^{i\phi_{s,21}}, \tag{S19}$$
$$h_k(\boldsymbol{r}_{2,i}, \boldsymbol{k}_{2,i}, \boldsymbol{r}_{0,i}, \boldsymbol{k}_{0,i}) = e^{i\phi_{i,20}}. \tag{S20}$$

The field correlation becomes

$$F = E_0^2 t_0 \sum_{\boldsymbol{k}'_{1,s}, \boldsymbol{k}_{2,s}} h_k(\boldsymbol{r}_{2,s}, \boldsymbol{k}_{2,s}; \boldsymbol{r}_{1,s}, \boldsymbol{k}'_{1,s})$$
$$\times \sum_{\boldsymbol{k}_{0,s}, \boldsymbol{k}_{1,s}, \boldsymbol{k}_{2,i}} h_k(\boldsymbol{r}_{0,s}, \boldsymbol{k}_{0,s}; \boldsymbol{r}_{1,s}, \boldsymbol{k}_{1,s}) h_k(\boldsymbol{r}_{2,i}, \boldsymbol{k}_{2,i}, \boldsymbol{r}_{0,i}, \boldsymbol{k}_{0,i})$$
$$= E_0^2 t_0 h_s(\boldsymbol{r}_{2,s}; \boldsymbol{r}_{1,s}) h'_{\mathrm{ep}}(\boldsymbol{r}_{2,i}, \boldsymbol{r}_{0,s}, \boldsymbol{r}_{0,i}; \boldsymbol{r}_{1,s}). \tag{S21}$$

Above, we define the following functions:

$$h_s(\boldsymbol{r}_{2,s}; \boldsymbol{r}_{1,s}) = \sum_{\boldsymbol{k}'_{1,s}, \boldsymbol{k}_{2,s}} h_k(\boldsymbol{r}_{2,s}, \boldsymbol{k}_{2,s}; \boldsymbol{r}_{1,s}, \boldsymbol{k}'_{1,s}), \tag{S22}$$

$$h'_{\mathrm{ep}}(\boldsymbol{r}_{2,i}, \boldsymbol{r}_{0,s}, \boldsymbol{r}_{0,i}; \boldsymbol{r}_{1,s}) = \sum_{\boldsymbol{k}_{0,s}, \boldsymbol{k}_{1,s}, \boldsymbol{k}_{2,i}} h_k(\boldsymbol{r}_{0,s}, \boldsymbol{k}_{0,s}; \boldsymbol{r}_{1,s}, \boldsymbol{k}_{1,s}) h_k(\boldsymbol{r}_{2,i}, \boldsymbol{k}_{2,i}; \boldsymbol{r}_{0,i}, \boldsymbol{k}_{0,i})$$
$$= \sum_{\boldsymbol{k}_{0,s}, \boldsymbol{k}_{1,s}, \boldsymbol{k}_{2,i}} e^{i\phi_{s,10}} e^{i\phi_{i,20}} = \sum_{\boldsymbol{k}_{0,s}, \boldsymbol{k}_{1,s}, \boldsymbol{k}_{2,i}} e^{i\phi_{\mathrm{ep}}}, \tag{S23}$$

where $\phi_{\mathrm{ep}} = \phi_{s,10} + \phi_{i,20}$ is the equivalent phase shift leading to h'_{ep}. The subscript ep stands for entanglement pinhole.

We then substitute the field correlation into the single-photon pair (SPP) intensity correlation $G^{(2)}_{\mathrm{SPP}}$:

$$G_{\mathrm{SPP}}^{(2)}(\boldsymbol{r}_{2,s},\boldsymbol{r}_{2,i},\boldsymbol{r}_{0,s},\boldsymbol{r}_{0,i};\boldsymbol{r}_{1,s}) = |F|^2$$
$$= \left|E_0^2(\boldsymbol{r}_{0,s})t_0\right|^2 \left|h_s(\boldsymbol{r}_{2,s};\boldsymbol{r}_{1,s})\right|^2 \left|h'_{\mathrm{ep}}(\boldsymbol{r}_{2,i},\boldsymbol{r}_{0,s},\boldsymbol{r}_{0,i};\boldsymbol{r}_{1,s})\right|^2. \quad \text{(S24)}$$

The detectors detect true coincidence of a photon pair within the coincidence window (8 ns). Photon pairs from different positions of the BBO most likely fall into different coincidence windows and, hence, are considered incoherent (*74*). Integrating the contributions of photon pairs from different positions incoherently yields the multi-photon pair (MPP) intensity correlation $G_{\mathrm{MPP}}^{(2)}$:

$$G_{\mathrm{MPP}}^{(2)}(\boldsymbol{r}_{2,s},\boldsymbol{r}_{2,i};\boldsymbol{r}_{1,s}) = \int_S p_0(\boldsymbol{r}_{0,s}) G_{\mathrm{SPP}}^{(2)}(\boldsymbol{r}_{2,s},\boldsymbol{r}_{2,i},\boldsymbol{r}_{0,s},\boldsymbol{r}_{0,i};\boldsymbol{r}_{1,s}) d\boldsymbol{r}_{0,s}$$
$$= |t_0|^2 \left|h_s(\boldsymbol{r}_{2,s};\boldsymbol{r}_{1,s})\right|^2 \left|h_{\mathrm{ep}}(\boldsymbol{r}_{2,i};\boldsymbol{r}_{1,s})\right|^2. \quad \text{(S25)}$$

Here, S represents the spatial domain of the BBO source, and $p_0(\boldsymbol{r}_{0,s})$ denotes the probability density function for each photon pair at the source position $\boldsymbol{r}_{0,s} = \boldsymbol{r}_{0,i}$. We define the following function to represent the entanglement pinhole effect:

$$\left|h_{\mathrm{ep}}(\boldsymbol{r}_{2,i};\boldsymbol{r}_{1,s})\right|^2 = \int_S p_0(\boldsymbol{r}_{0,s}) \left|E_0^2(\boldsymbol{r}_{0,s}) h'_{\mathrm{ep}}(\boldsymbol{r}_{2,i},\boldsymbol{r}_{0,s},\boldsymbol{r}_{0,i};\boldsymbol{r}_{1,s})\right|^2 d\boldsymbol{r}_{0,s}. \quad \text{(S26)}$$

If the p_0 and E_0 distributions are broad and S is large, the integral reduces to wide-field illumination:

$$\left|h_{\mathrm{ep}}(\boldsymbol{r}_{2,i};\boldsymbol{r}_{1,s})\right|^2 \propto \int_S \left|h'_{\mathrm{ep}}(\boldsymbol{r}_{2,i},\boldsymbol{r}_{0,s},\boldsymbol{r}_{0,i};\boldsymbol{r}_{1,s})\right|^2 d\boldsymbol{r}_{0,s}, \quad \text{(S27)}$$

which represents the PSF on $\boldsymbol{r}_{2,i}$ at a given $\boldsymbol{r}_{1,s}$, treating the BBO crystal source as a mirror. Therefore, while the two detectors provide spatial resolution, the source provides none.

Integration over the finite apertures of the two detectors yields the final intensity correlation $G_{\mathrm{ICE}}^{(2)}$:

$$G_{\mathrm{ICE}}^{(2)}(\boldsymbol{r}_{1,s}) = |t_0|^2 \int_{D_s} p_s(\boldsymbol{r}_{2,s}) \left|h_s(\boldsymbol{r}_{2,s};\boldsymbol{r}_{1,s})\right|^2 d\boldsymbol{r}_{2,s}$$
$$\times \int_{D_i} p_i(\boldsymbol{r}_{2,i}) \left|h_{\mathrm{ep}}(\boldsymbol{r}_{2,i};\boldsymbol{r}_{1,s})\right|^2 d\boldsymbol{r}_{2,i}, \quad \text{(S28)}$$

where $p_s(\boldsymbol{r}_{2,s})$ and $p_i(\boldsymbol{r}_{2,i})$ denote the photon detection probability density functions for the two detectors, respectively.

For classical imaging (CI) using signal-channel-only detection (i.e., raw singles photon counts received by D_s), the wavefunction of the signal photon emitted from $\boldsymbol{r}_{0,s}$ is

$$|\xi_s\rangle = \sum_{\mathbf{k}_{0,s}} e^{-j\mathbf{k}_{0,s}\cdot\mathbf{r}_{0,s}} \left|1_{\mathbf{k}_{0,s}}\right\rangle. \quad \text{(S29)}$$

With $\hat{E}_s^{(+)}$and $|\xi_s\rangle$ we find the received field as

$$F_s = \left\langle 0_{\boldsymbol{r}_{2,s}}\right| \hat{E}_s^{(+)}(\boldsymbol{r}_{2,s},\boldsymbol{r}_{0,s};\boldsymbol{r}_{1,s}) |\xi_s\rangle$$
$$= E_0(\boldsymbol{r}_{0,s})\hat{e} \sum_{\boldsymbol{k}_{0,s},\boldsymbol{k}_{1,s},\boldsymbol{k}'_{1,s},\boldsymbol{k}_{2,s}} t_0 h_k(\boldsymbol{r}_{2,s},\boldsymbol{k}_{2,s};\boldsymbol{r}_{1,s},\boldsymbol{k}'_{1,s}) h_k(\boldsymbol{r}_{0,s},\boldsymbol{k}_{0,s};\boldsymbol{r}_{1,s},\boldsymbol{k}_{1,s}). \quad \text{(S30)}$$

Hence, we derive the total intensity $G_{\mathrm{CI}}^{(1)}(\boldsymbol{r}_{2,s};\boldsymbol{r}_{1,s})$ to be

$$G_{\mathrm{CI}}^{(1)}(\boldsymbol{r}_{1,s}) = \int_{D_s} p_s(\boldsymbol{r}_{2,s}) d\boldsymbol{r}_{2,s} \int_S p_0(\boldsymbol{r}_{0,s}) |F_s|^2 d\boldsymbol{r}_{0,s}$$
$$= |t_0|^2 \int_{D_s} p_s(\boldsymbol{r}_{2,s}) \left|h_s(\boldsymbol{r}_{2,s};\boldsymbol{r}_{1,s})\right|^2 d\boldsymbol{r}_{2,s}$$
$$\times \int_S p_0(\boldsymbol{r}_{0,s}) \left|E_0(\boldsymbol{r}_{0,s}) h_s(\boldsymbol{r}_{0,s};\boldsymbol{r}_{1,s})\right|^2 d\boldsymbol{r}_{0,s}$$
$$, \tag{S31}$$

Where $h_s(\boldsymbol{r}_{0,s};\boldsymbol{r}_{1,s})$ and $h_s(\boldsymbol{r}_{2,s};\boldsymbol{r}_{1,s})$ are the PSFs. If the p_0 and E_0 distributions are broad and S is large, the second integral approaches a constant as in wide-field illumination. Therefore, only the signal-arm detector provides spatial resolution while the source provides none:

$$G_{\mathrm{CI}}^{(1)}(\boldsymbol{r}_{1,s}) \propto |t_0|^2 \int_{D_s} p_s(\boldsymbol{r}_{2,s}) \left|h_s(\boldsymbol{r}_{2,s};\boldsymbol{r}_{1,s})\right|^2 d\boldsymbol{r}_{2,s}\,. \tag{S32}$$

From Eq. (S28) and Eq. (S32), we have

$$G_{\mathrm{ICE}}^{(2)}(\boldsymbol{r}_{1,s}) \propto G_{\mathrm{CI}}^{(1)}(\boldsymbol{r}_{1,s}) \times G_{\mathrm{ep}}^{(1)}(\boldsymbol{r}_{1,s}), \tag{S33}$$

where $G_{\mathrm{ep}}^{(1)} = \int_{D_i} p_i(\boldsymbol{r}_{2,i}) \left|h_{\mathrm{ep}}(\boldsymbol{r}_{2,i};\boldsymbol{r}_{1,s})\right|^2 d\boldsymbol{r}_{2,i}$ represents the entanglement pinhole effect. The idler-arm detector functions as a virtual pinhole for ICE, enhancing the classical resolution. If the idler-arm detector becomes an infinitely large bucket detector, then $G_{\mathrm{ICE}}^{(2)}(\boldsymbol{r}_{1,s}) \propto G_{\mathrm{CI}}^{(1)}(\boldsymbol{r}_{1,s})$, i.e., ICE approaches CI. Conversely, if both detectors are point-like, we have asymptotically $G_{\mathrm{ICE}}^{(2)}(\boldsymbol{r}_{1,s}) \propto \left|h_s(\boldsymbol{r}_{2,s}=0;\boldsymbol{r}_{1,s})\right|^2 \times \left|h_{\mathrm{ep}}(\boldsymbol{r}_{2,i}=0;\boldsymbol{r}_{1,s})\right|^2$.

Note S3 ICE using accidental coincidences

If the two detectors only measure accidental coincidences, the results could be treated as coincidences of photons from different positions $(\boldsymbol{r}_{0,s},\boldsymbol{r}_{0,i})$ with different wavevectors $(\boldsymbol{k}_{0,s},\boldsymbol{k}_{0,i})$. The source state is replaced by

$$|\xi\rangle_{\mathrm{acc}} = \sum_{\boldsymbol{k}_{0,s},\boldsymbol{k}_{0,i}} e^{-j\boldsymbol{k}_{0,s}\cdot\boldsymbol{r}_{0,s}} e^{-j\boldsymbol{k}_{0,i}\cdot\boldsymbol{r}_{0,i}} \left|1_{\boldsymbol{k}_{0,s}},1_{\boldsymbol{k}_{0,i}}\right\rangle, \tag{S34}$$

which can be written as a product state $|\xi\rangle_{\mathrm{acc}} = \sum_{\boldsymbol{k}_{0,s}} e^{-j\boldsymbol{k}_{0,s}\cdot\boldsymbol{r}_{0,s}} \left|1_{\boldsymbol{k}_{0,s}}\right\rangle \otimes \sum_{\boldsymbol{k}_{0,i}} e^{-j\boldsymbol{k}_{0,i}\cdot\boldsymbol{r}_{0,i}} \left|1_{\boldsymbol{k}_{0,i}}\right\rangle$. The intensity correlation related to accidental coincidences is

$$G_{\mathrm{acc}}^{(2)}(\boldsymbol{r}_{2,s},\boldsymbol{r}_{2,i};\boldsymbol{r}_{1,s})$$
$$= |t_0|^2 \left|\sum_{\boldsymbol{k}'_{1,s},\boldsymbol{k}_{2,s}} h_k(\boldsymbol{r}_{2,s},\boldsymbol{k}_{2,s};\boldsymbol{r}_{1,s},\boldsymbol{k}'_{1,s})\right|^2$$
$$\times \int_S p_0(\boldsymbol{r}_{0,s}) \left|E_0(\boldsymbol{r}_{0,s}) \sum_{\boldsymbol{k}_{0,s},\boldsymbol{k}_{1,s}} h_k(\boldsymbol{r}_{0,s},\boldsymbol{k}_{0,s};\boldsymbol{r}_{1,s},\boldsymbol{k}_{1,s})\right|^2 d\boldsymbol{r}_{0,s}$$
$$\times \int_S p_0(\boldsymbol{r}_{0,i}) \left|E_0(\boldsymbol{r}_{0,i}) \sum_{\boldsymbol{k}_{0,i},\boldsymbol{k}_{2,i}} h_k(\boldsymbol{r}_{2,i},\boldsymbol{k}_{2,i},\boldsymbol{r}_{0,i},\boldsymbol{k}_{0,i})\right|^2 d\boldsymbol{r}_{0,i}\,. \tag{S35}$$

Next, we can use $h_s(\boldsymbol{r}_{2,s};\boldsymbol{r}_{1,s}) = \sum_{\boldsymbol{k}'_{1,s},\boldsymbol{k}_{2,s}} h_k(\boldsymbol{r}_{2,s},\boldsymbol{k}_{2,s};\boldsymbol{r}_{1,s},\boldsymbol{k}'_{1,s})$, $h_s(\boldsymbol{r}_{0,s};\boldsymbol{r}_{1,s}) =$

$\sum_{\boldsymbol{k}_{0,s},\boldsymbol{k}_{1,s}} h_k(\boldsymbol{r}_{1,s},\boldsymbol{k}_{1,s};\boldsymbol{r}_{0,s},\boldsymbol{k}_{0,s})$, and $h_i(\boldsymbol{r}_{2,i},\boldsymbol{r}_{0,i}) = \sum_{\boldsymbol{k}_{2,i},\boldsymbol{k}_{0,i}} h_k(\boldsymbol{r}_{2,i},\boldsymbol{k}_{2,i},\boldsymbol{r}_{0,i},\boldsymbol{k}_{0,i})$ to simplify $G_{\mathrm{acc}}^{(2)}$:

$$G_{\mathrm{acc}}^{(2)}(\boldsymbol{r}_{1,s};\boldsymbol{r}_{2,s},\boldsymbol{r}_{2,i}) = |t_0|^2 \left|h_s(\boldsymbol{r}_{2,s};\boldsymbol{r}_{1,s})\right|^2 \int_S p_0(\boldsymbol{r}_{0,s}) \left|E_0(\boldsymbol{r}_{0,s}) h_s(\boldsymbol{r}_{0,s};\boldsymbol{r}_{1,s})\right|^2 d\boldsymbol{r}_{0,s}$$
$$\times \int_S p_0(\boldsymbol{r}_{0,i}) \left|E_0(\boldsymbol{r}_{0,i}) h_i(\boldsymbol{r}_{2,i},\boldsymbol{r}_{0,i})\right|^2 d\boldsymbol{r}_{0,i}\,. \qquad (S36)$$

Integration over the finite aperture of the detector yields

$$G_{\mathrm{acc}}^{(2)}(\boldsymbol{r}_{1,s}) = |t_0|^2 \int_{D_s} p_s(\boldsymbol{r}_{2,s}) \left|h_s(\boldsymbol{r}_{2,s};\boldsymbol{r}_{1,s})\right|^2 d\boldsymbol{r}_{2,s} \int_S p_0(\boldsymbol{r}_{0,s}) \left|E_0(\boldsymbol{r}_{0,s}) h_s(\boldsymbol{r}_{0,s};\boldsymbol{r}_{1,s})\right|^2 d\boldsymbol{r}_{0,s}$$
$$\times \int_{D_i} \int_S p_i(\boldsymbol{r}_{2,i}) p_0(\boldsymbol{r}_{0,i}) \left|E_0(\boldsymbol{r}_{0,i}) h_i(\boldsymbol{r}_{2,i},\boldsymbol{r}_{0,i})\right|^2 d\boldsymbol{r}_{0,i}\, d\boldsymbol{r}_{2,i}\,. \qquad (S37)$$

Because the last term is a constant with a determined D_i and S, comparing Eq. (S37) with Eq. (S31), we have

$$G_{\mathrm{acc}}^{(2)}(\boldsymbol{r}_{1,s}) \propto G_{\mathrm{CI}}^{(1)}. \qquad (S38)$$

Imaging with accidental coincidences therefore provides the same resolution and DOF as classical imaging.

Note S4 Characterization of polarization entanglement through Bell's test

Bell-type inequalities provide a standard to characterize a system's ability to generate entangled states. These inequalities are constructed in favor of the local hidden variable theory (LHVT), while the violation of them as predicted by quantum mechanics is generally observed in experiments. Among the various means of Bell's tests, the Clauser–Horne–Shimony–Holt (CHSH) inequality serves as a practical benchmark (*58*).

Defining the Hilbert space for Alice as $\mathcal{A}$ and for Bob as $\mathcal{B}$, we denote eigenstates for the measurement axes $\hat{\alpha}$ and $\hat{\beta}$ as A_α and B_β, and the corresponding eigenvalues as a and b. An LHVT suggests a hidden variable λ with a probability density function $p(\lambda)$. For measurement outcomes a and b, their joint probability is

$$P_{\mathrm{LHV}}(a,b|\alpha,\beta) = \int P(a|\alpha,\lambda) P(b|\beta,\lambda) p(\lambda) d\lambda, \qquad (S39)$$

where $P(a|\alpha,\lambda)$ and $P(b|\beta,\lambda)$ are probabilities for Alice to obtain a and Bob to obtain b, respectively. Now Alice and Bob set two analyzers with random angles of α and β. $E(\alpha,\beta)$ represents the correlation of the measurement:

$$E(\alpha,\beta) \equiv P(H,H|\alpha,\beta) + P(V,V|\alpha,\beta) - P(H,V|\alpha,\beta) - P(V,H|\alpha,\beta). \qquad (S40)$$

The Bell–CHSH inequality is then given by

$$S_{\mathrm{CHSH}} = |E(\alpha,\beta) + E(\alpha',\beta) - E(\alpha,\beta') + E(\alpha',\beta')| \leq 2, \qquad (S41)$$

where α' and β' denote the second choices of the analyzer angles.

In contrast, quantum theory predicts that $S_{\mathrm{CHSH}} > 2$ is possible with specific combinations of observation angles. According to quantum mechanics, we can model coincidence counts $\widehat{N}(H,H|\alpha,\beta)$ for a maximally entangled Bell state (i.e., the EPR state) as (*57*)

$$\widehat{N}(H,H|\alpha,\beta) = N_0 \cos^2(\alpha-\beta) + N_1, \qquad (S42)$$

where N_0 is the maximum true coincidence count, and N_1 represents the contribution of accidental coincidences. For such states, quantum theory predicts a maximum violation of Eq. (S41) at

$(0°, 22.5°, 45°, 67.5°)$ at the Tsirelson's bound, $S_{\mathrm{CHSH}}^{\max} = 2\sqrt{2}$.

In our experimental setup, for each round of Bell's test, we rotated α from 0° to 180° with a step size of 45°. For each fixed α, we rotated β from 0° to 180° with a step size of 22.5°. After recording the coincidence counts $N(H,H|\alpha,\beta)$ at each step with an acquisition time of 1 s and a coincidence detection window of 8 ns, we calculated the correlation value adapted from Eq. (S40) as

$$E(\alpha,\beta) = \frac{N(H,H|\alpha,\beta)+N(H,H|\alpha+90°,\beta+90°)-N(H,H|\alpha+90°,\beta)-N(H,H|\alpha,\beta+90°)}{N(H,H|\alpha,\beta)+N(H,H|\alpha+90°,\beta+90°)+N(H,H|\alpha+90°,\beta)+N(H,H|\alpha,\beta+90°)}. \tag{S43}$$

The CHSH S value was then evaluated based on the value of E according to Eq. (S41). The results are shown in Fig. S10.

By performing Bell's test, our system shows a strong violation of the CHSH inequality with $S = 2.78 \pm 0.01 > 2$ estimated by calculating the mean and standard error of S values measured from 10 rounds of Bell's tests. This result, which violates the Bell-CHSH inequality by more than 57 standard errors of the mean, indicates substantial deviations of our result from the LHVT prediction.

Note S5 Polarization entanglement-enabled quantitative quantum birefringence imaging

The polarization entanglement of the SPDC photons in the ICE system enables quantitative quantum birefringence imaging. By preparing the EPR state as described in Eq. (S42) and recording the coincidence counts, ICE can be used to quantify the transmittance T and the full birefringence properties θ and Δ of the object, where θ is the angle of the principal refractive index axis and Δ is the phase retardation between the two refractive index axes. Here, we use Stokes vectors (I, Q, U, V) and Mueller matrices to describe the state of polarization. The birefringence properties of the object can be denoted using a Mueller matrix (*48*):

$$X_{\Delta,\theta} = \begin{bmatrix} 1 & 0 & 0 & 0 \\ 0 & \cos^2 2\theta + \sin^2 2\theta \cos\Delta & \cos 2\theta \sin 2\theta (1-\cos\Delta) & -\sin 2\theta \sin\Delta \\ 0 & \cos 2\theta \sin 2\theta (1-\cos\Delta) & \sin^2 2\theta + \cos^2 2\theta \cos\Delta & \cos 2\theta \sin\Delta \\ 0 & \sin 2\theta \sin\Delta & -\cos 2\theta \sin\Delta & \cos\Delta \end{bmatrix}. \tag{S44}$$

Under the EPR state, we kept $\alpha = 0°$ in the signal arm while rotating β in the idler arm from 0° to 135° with a step size of 45°. The corresponding polarization states of the coincidence measurements can be represented by the Stokes vectors

$$\vec{S}_{\beta=0°} = \begin{bmatrix} I_{0°} \\ Q_{0°} \\ U_{0°} \\ V_{0°} \end{bmatrix} = X_{\Delta,\theta} \begin{bmatrix} 1 \\ 1 \\ 0 \\ 0 \end{bmatrix} T, \tag{S45}$$

$$\vec{S}_{\beta=45°} = \begin{bmatrix} I_{45°} \\ Q_{45°} \\ U_{45°} \\ V_{45°} \end{bmatrix} = X_{\Delta,\theta} \begin{bmatrix} 1 \\ 0 \\ 1 \\ 0 \end{bmatrix} T, \tag{S46}$$

$$\vec{S}_{\beta=90°} = \begin{bmatrix} I_{90°} \\ Q_{90°} \\ U_{90°} \\ V_{90°} \end{bmatrix} = X_{\Delta,\theta} \begin{bmatrix} 1 \\ -1 \\ 0 \\ 0 \end{bmatrix} T, \tag{S47}$$

$$\vec{S}_{\beta=135^\circ} = \begin{bmatrix} I_{135^\circ} \\ Q_{135^\circ} \\ U_{135^\circ} \\ V_{135^\circ} \end{bmatrix} = X_{\Delta,\theta} \begin{bmatrix} 1 \\ 0 \\ -1 \\ 0 \end{bmatrix} T. \tag{S48}$$

Substituting Eq. (S44) into Eqs. (S45)–(S48), we have

$$I_{0^\circ} = T, Q_{0^\circ} = T(\cos^2 2\theta + \sin^2 2\theta \cos\Delta),$$
$$I_{45^\circ} = T,\ Q_{45^\circ} = T\cos 2\theta \sin 2\theta (1 - \cos\Delta),$$
$$I_{90^\circ} = T, Q_{90^\circ} = -T(\cos^2 2\theta + \sin^2 2\theta \cos\Delta),$$
$$I_{135^\circ} = T,\ Q_{135^\circ} = -T\cos 2\theta \sin 2\theta (1 - \cos\Delta).$$

The coincidence counts for $\beta = 0°, 45°, 90°, 135°$, therefore, can be represented as

$$N_{0^\circ} = \frac{1}{2}(I_{0^\circ} + Q_{0^\circ}) = \frac{T}{2}(1 + \cos^2 2\theta + \sin^2 2\theta \cos\Delta), \tag{S49}$$

$$N_{45^\circ} = \frac{1}{2}(I_{45^\circ} + Q_{45^\circ}) = \frac{T}{2}(1 + \cos 2\theta \sin 2\theta (1 - \cos\Delta)), \tag{S50}$$

$$N_{90^\circ} = \frac{1}{2}(I_{90^\circ} + Q_{90^\circ}) = \frac{T}{2}(1 - \cos^2 2\theta - \sin^2 2\theta \cos\Delta), \tag{S51}$$

$$N_{135^\circ} = \frac{1}{2}(I_{135^\circ} + Q_{135^\circ}) = \frac{T}{2}(1 - \cos 2\theta \sin 2\theta (1 - \cos\Delta)). \tag{S52}$$

Consequently, based on Eqs. (S49)–(S52), the transmittance and full birefringence properties of the object can be extracted from the coincidence counts:

$$T = \frac{1}{2}(N_{0^\circ} + N_{45^\circ} + N_{90^\circ} + N_{135^\circ}), \tag{S53}$$

$$\theta = \frac{1}{2}\tan^{-1}\left(\frac{2N_{90^\circ}}{N_{45^\circ} - N_{135^\circ}}\right), \tag{S54}$$

$$\Delta = \cos^{-1}\left(1 - \frac{(N_{45^\circ} - N_{135^\circ})^2 + 4N_{90^\circ}^2}{N_{90^\circ}(N_{0^\circ} + N_{45^\circ} + N_{90^\circ} + N_{135^\circ})}\right). \tag{S55}$$

In classical imaging, the birefringence properties of the object need to be measured using incident photons with different polarization states (*75*). In ICE, however, quantitative birefringence imaging can be performed without changing the polarization states of the photons incident on the object. When the polarization of the signal photons was kept constant ($\alpha = 0°$) while the polarization states of the idler photons were varied ($\beta = 0°, 45°, 90°, 135°$), the classical images acquired with the raw signal counts showed no differences, unable to extract the birefringence properties of the object (Fig. S11A). In comparison, the ICE images acquired with the coincidence counts exhibited substantial differences following Eqs. (S49)–(S52) (Fig. S11B), which could be used to extract the transmittance and full birefringence properties of the object (Fig. S11, C–E). One may regard this approach as quantum "ghost birefringence quantification". Enabled by polarization entanglement, the ghost birefringence quantification of ICE demonstrates a true quantum advantage over classical imaging.

Note S6 Imaging by coincidence with a classical light source

Classical two-photon coincidence imaging can be achieved when the spatially entangled source is replaced with a classical pulse source (*59*). We implemented such imaging as shown in Fig. S13, where a 635-nm CW laser (MLL-III-635-100mW, CNI Laser) was modulated at 4 kHz by a mechanical chopper (MC1F60, Thorlabs). The modulated beam was split by a beam splitter (BS013, Thorlabs) and sent to the signal and idler arms of the ICE system in Fig. 1A, where the

polarization selectors (HWP and PBS) were removed. The images formed with the raw signal counts using the quantum and classical sources are shown in Fig. S14A and B, respectively. For a fair comparison, we used a neutral density filter to attenuate the classical beam such that it provided the same photon flux to the object as that of the SPDC signal beam (~19k photons per second at maximum transmittance). Because the SPDC beam contained more spatial modes than the classical beam, the raw signal image (i.e., the classical image) generated with the quantum source exhibited a lower spatial resolution than the one generated with the classical source.

Using the quantum source, the ICE image in Fig. S14C showed a maximum coincidence count rate at 485 Hz with an SNR of 22. In comparison, using the classical source, the ICE image in Fig. S14D exhibited a maximum coincidence count rate at 16 Hz with an SNR of 4, which was 5.5 times lower than the SNR from the quantum source. Therefore, whereas it is possible to use classical correlation to generate ICE images, the SNR of the image is substantially lower than that generated with a spatially entangled quantum source. Consequently, to generate ICE images with the same SNR, the object needs to be illuminated with a classical source that is 30 times stronger than that of a quantum source, which could cause damage to photosensitive biological samples. Moreover, classical correlation is incompatible with either the sub-shot-noise algorithms (Note S1), which require a spatially entangled quantum source, or the ghost birefringence quantification that is enabled by polarization entanglement (Note S5). Therefore, the quantum correlation of hyperentangled SPDC photons in ICE is advantageous over classical correlation, especially for imaging photosensitive biological samples.

Note S7 Comparison of ICE, GI, and CPI

Here, we compare ICE (Fig. S15A) with two existing quantum imaging methods. Quantum ghost imaging (GI) utilizes spatially entangled photons to record an image of an object using photons that have not interacted with the object (*62*). A typical ghost imaging setup uses entangled SPDC photons generated by a nonlinear medium, e.g., a BBO crystal, and exploits the spatial correlations between the positions of the photon pairs in the signal and idler arms (Fig. S15B). The signal photons interact with the object and is detected by a non-spatially resolving bucket detector. The idler photons are detected by a multi-pixel camera, which, upon coincidence detection of signal and idler photons, provides a ghost image of the object. It is noted that neither signal nor idler beams alone contain enough information to reconstruct an image of the object. However, the spatial entanglement between the signal and idler photons can be utilized to extract the image.

As an extension of GI, correlation plenoptic imaging (CPI) is another quantum imaging method that utilizes spatial correlations of photon pairs (*63*, *64*). Beyond the position correlation utilized in GI, CPI also exploits the momentum correlation of the photon pairs, capturing the light field (position and direction of the light) emanating from the object, thus allowing refocusing, DOF extension, and 3D visualization. Unlike GI, which requires a bucket detector and a camera, CPI utilizes two well-aligned multi-pixel cameras to simultaneously record the position and momentum of the photon pairs. CPI has been demonstrated experimentally with chaotic light (*76*) and entangled SPDC photons (*64*, *77*). A framework of CPI with entangled photons adopts a multi-pixel camera in the idler arm to record the positions of the object, which, upon coincidence detection with the camera in the signal arm, provides a ghost image of the object (Fig. S15C). Additionally, through a lens that conjugates the BBO crystal and the camera in the signal arm, the momentum of the photons at each pixel of the object is recorded. The simultaneous recording of

the position and momentum of the spatially entangled photons enables the reconstruction of a plenoptic image of the object.

Despite the similarity in using entangled photon pairs and coincidence detection for imaging, ICE is fundamentally different from GI or CPI (Fig. S15). First, ICE directly images the object by focusing the SPDC beam onto the object and recording the coincidence of two single-pixel detectors (SPCMs) while raster scanning the object. The raster scanning conveniently extends the resolvable pixel counts, enabling quantum imaging over a large FOV. In comparison, both GI and most CPI techniques provide indirect ghost images of the object by triggering a multi-pixel camera using either a bucket detector or another camera. Because the multi-pixel cameras have a limited number of resolvable pixel counts, the FOVs of GI and CPI are limited. Second, ICE provides spatial resolution of the object, so it is capable of classically imaging the object using the signal arm alone. GI and most CPI techniques, however, do not provide spatial resolution of the object, and hence cannot image the object by only using the signal arm. Third, owing to the focusing and single-pixel detection of the SPDC beam, ICE measures substantially more spatial modes per pixel than GI and CPI, where the modes of the SPDC beam are distributed across the multi-pixel cameras. The larger number of spatial modes per pixel leads to a higher SNR of ICE over GI or CPI under the same photon flux. Fourth, whereas ICE, GI, and CPI all utilize only spatially entangled photon pairs generated from BBO crystals, ICE also exploits the polarization entanglement of the photon pairs for quantitative quantum birefringence imaging of the object (Note S5).

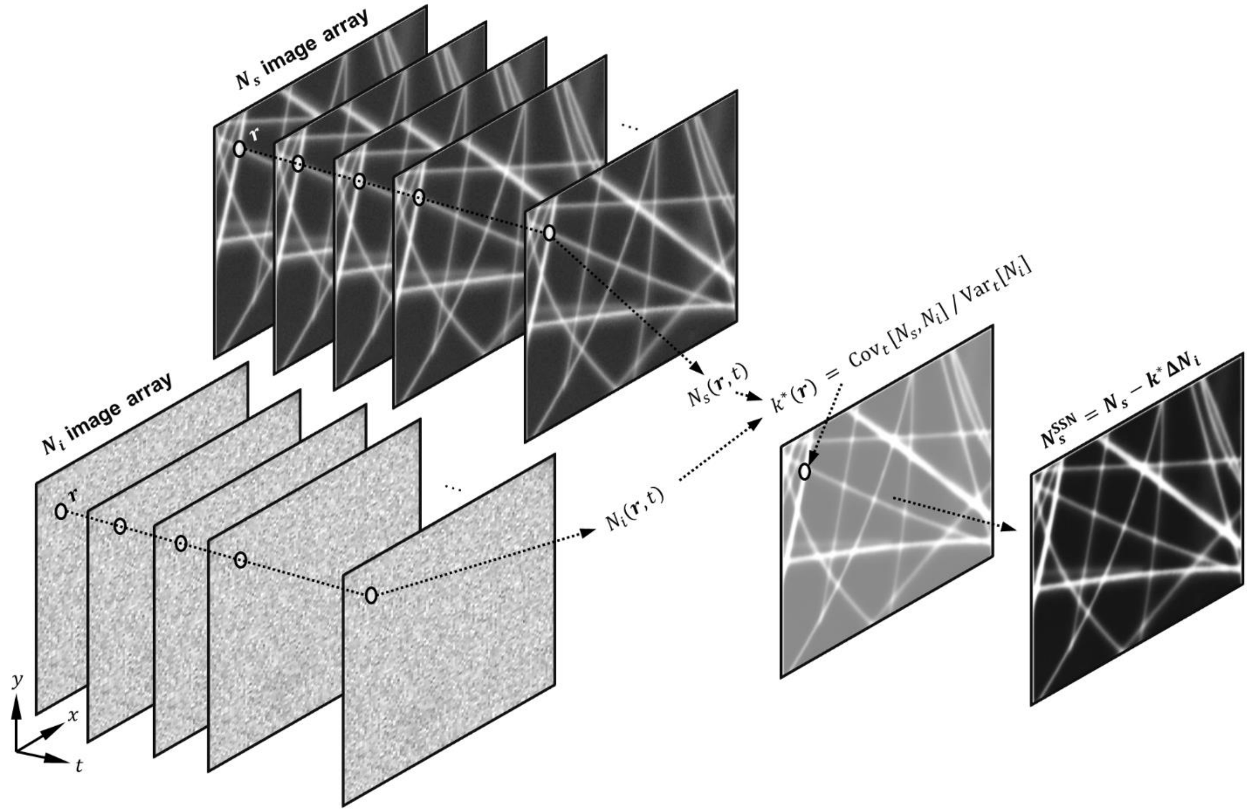

Fig. S1 Illustration of the CoV algorithm.

Each pixel $\boldsymbol{r}$ from the image stacks $N_s(\boldsymbol{r},t)$ and $N_i(\boldsymbol{r},t)$ form two time sequences, and their temporal covariance and variance are computed to generate $k^*(\boldsymbol{r})$ using Eq. (S7). The $k^*(\boldsymbol{r})$ image is used in the CoV algorithm to form the sub-shot-noise image $N_s^{\mathrm{SSN}}(\boldsymbol{r})$ using Eq. (S8).

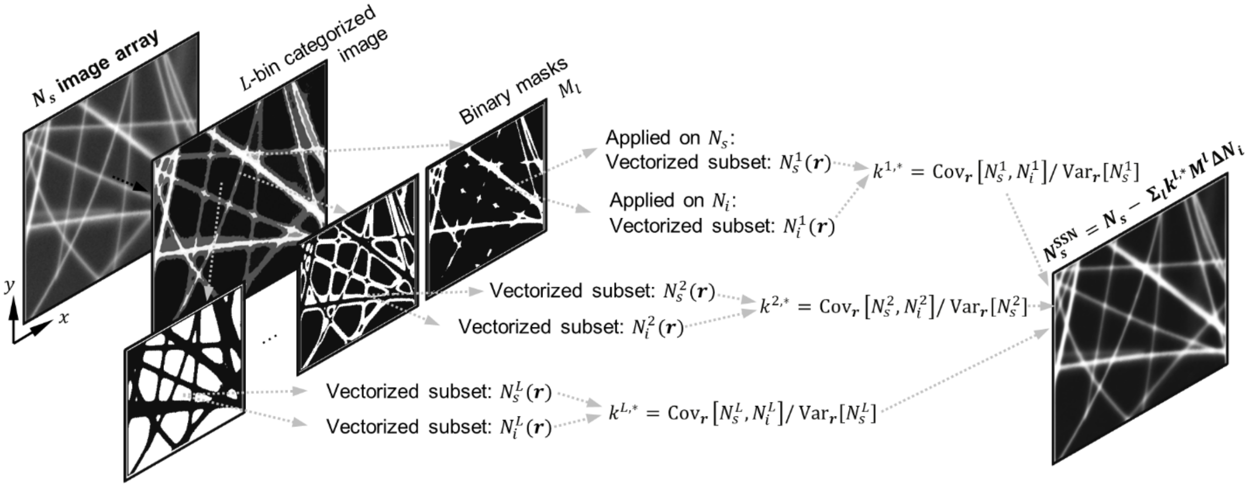

Fig. S2 Illustration of the s-CoV algorithm.

The images $N_s(\boldsymbol{r})$ and $N_i(\boldsymbol{r})$ are divided into L subset image pairs according to the pixel values in the N_s image. For each image pair N_s^l and N_i^l, the $k^{l,*}$ value is computed through Eq. (S10). The same procedure is repeated for all image pairs to form the $k^*(\boldsymbol{r})$ image, which is used in the s-CoV algorithm to form the sub-shot-noise image $N_s^{\mathrm{SSN}}(\boldsymbol{r})$ using Eq. (S12).

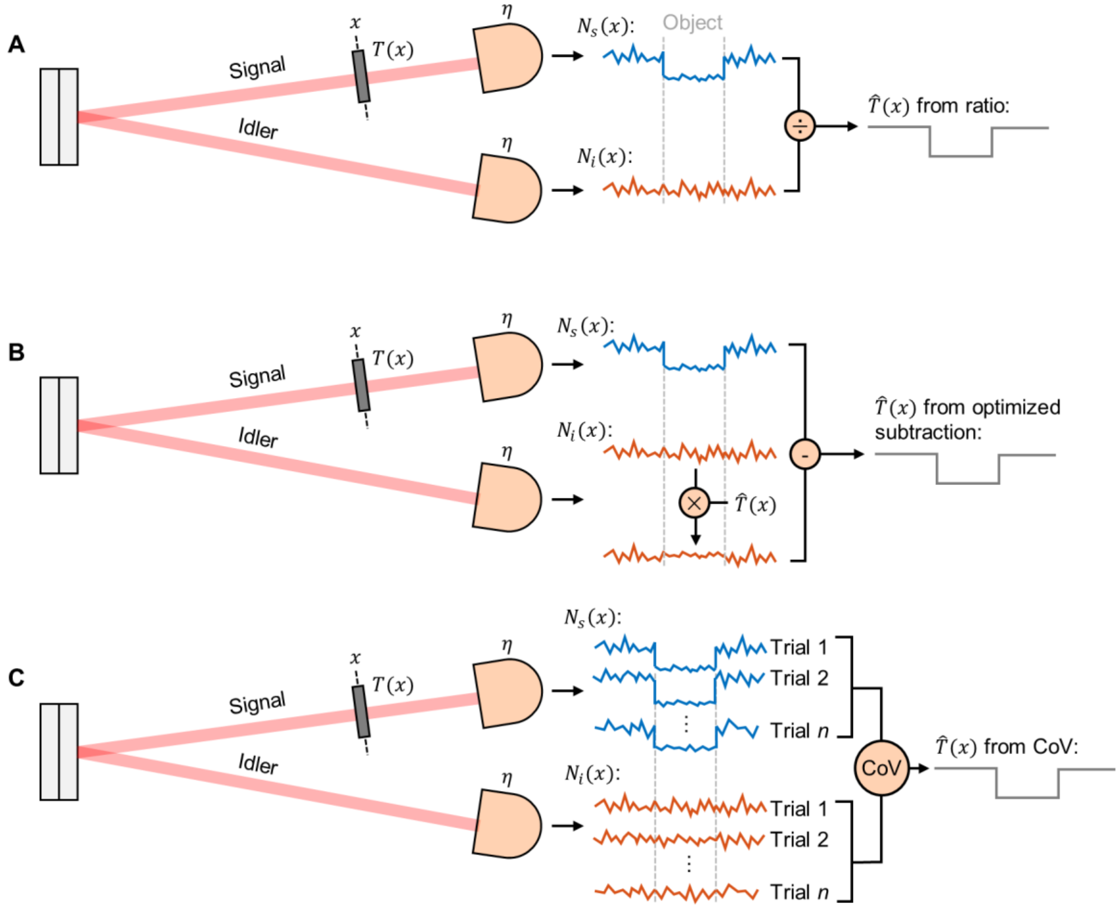

Fig. S3 Comparison of the workflows of the CoV and existing SSN algorithms.
(**A**) Workflow of the ratio (Eq. (S2)) algorithm. (**B**) Workflow of the optimized subtraction (Eq. (S4)) algorithm. (**C**) Workflow of the CoV (Eq. (S8)) algorithm.

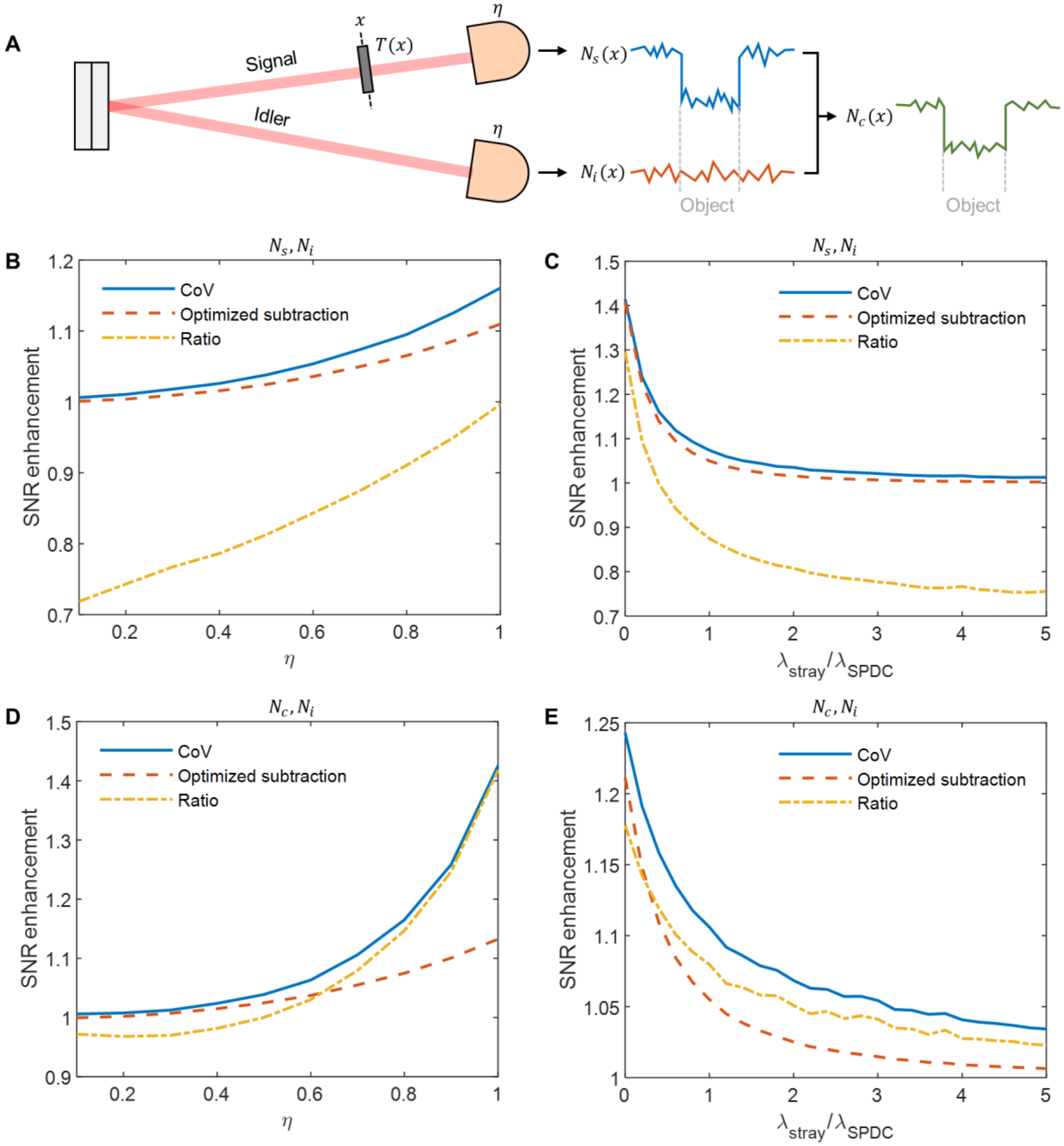

Fig. S4 SSN signal retrieval simulations.

(**A**) Schematics of the simulated setup. 1D object with transmittance $T(x) = 0.5$ is placed in the signal arm. $N_s(x)$ and $N_i(x)$ denote the images measured by detectors in the signal and idler arms. η is the detection efficiency. The $N_c(x)$ image is extracted from N_s and N_i. (**B**) SNR enhancement over classical measurement (Eq. (S1)) simulated using the ratio (Eq. (S2)), optimized subtraction (Eq. (S4)), and CoV (Eq. (S8)) algorithms with N_s and N_i. The ratio of stray light and SPDC photon counts is fixed to 1, and the detector efficiency η is varied. (**C**) Simulated SNR enhancement from N_s and N_i with the detector efficiency η fixed at 0.7 and the ratio of stray light and SPDC photon counts varied. (**D**) Simulated SNR enhancement from N_c and N_i with the ratio of stray light and SPDC photon counts fixed to 1 and the detector efficiency η varied. (**E**) Simulated SNR enhancement from N_c and N_i with the detector efficiency η fixed at 0.7 and the ratio of stray light and SPDC photon counts varied.

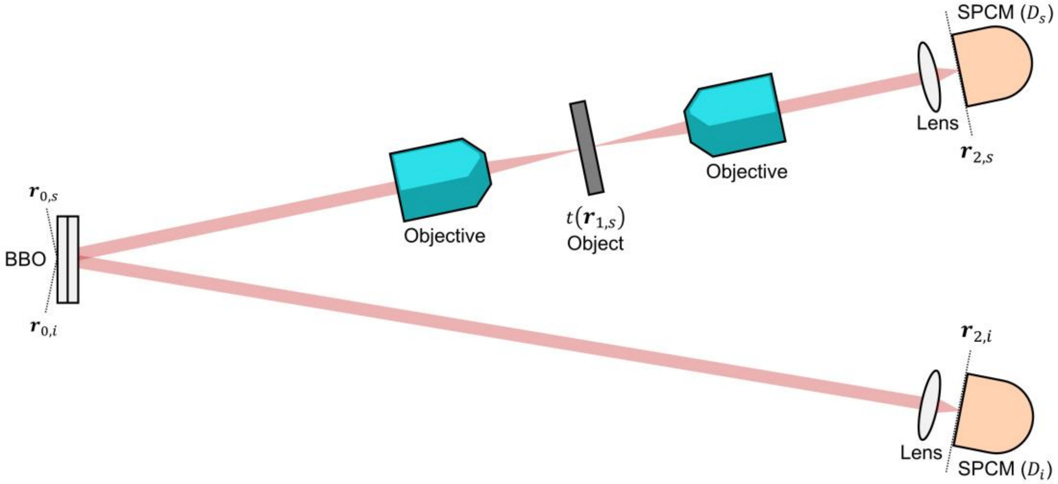

Fig. S5 Illustration of the ICE model.

SPCM, single photon counting module. $\boldsymbol{r}_{0,s}$, signal photon position on the source. $\boldsymbol{r}_{0,i}$, idler photon position on the source. D_s, D_i, detectors. $t(\boldsymbol{r}_{1,s})$, amplitude transmission coefficient of the object. $\boldsymbol{r}_{1,s}$, the position on the object. $\boldsymbol{r}_{2,s}$ and $\boldsymbol{r}_{2,i}$, the positions on the detectors D_s and D_i, respectively.

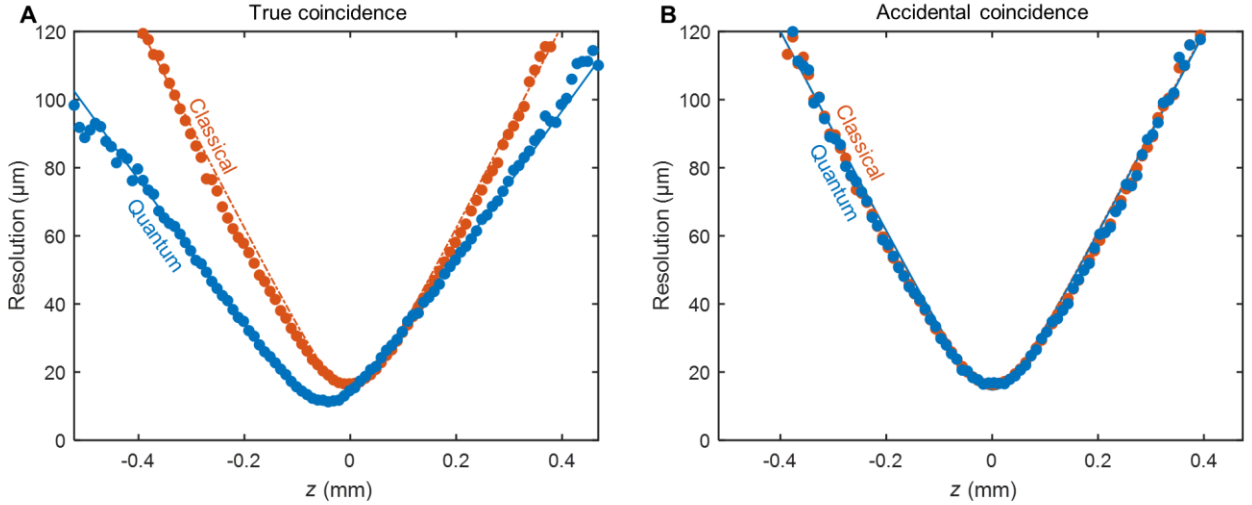

Fig. S6 ICE with true and accidental coincidences.
(**A** and **B**) Resolution versus *z* measured with true coincidences (A) and accidental coincidences (B). Dots represent experimental measurements. Solid and dash-dotted lines denote fits. The ICE measurements require the coincidence window to be 8 ns. For the accidental ICE measurements, the coincidence window was set to 400 ns to allow accidental coincidences. The results are explained in Note S3.

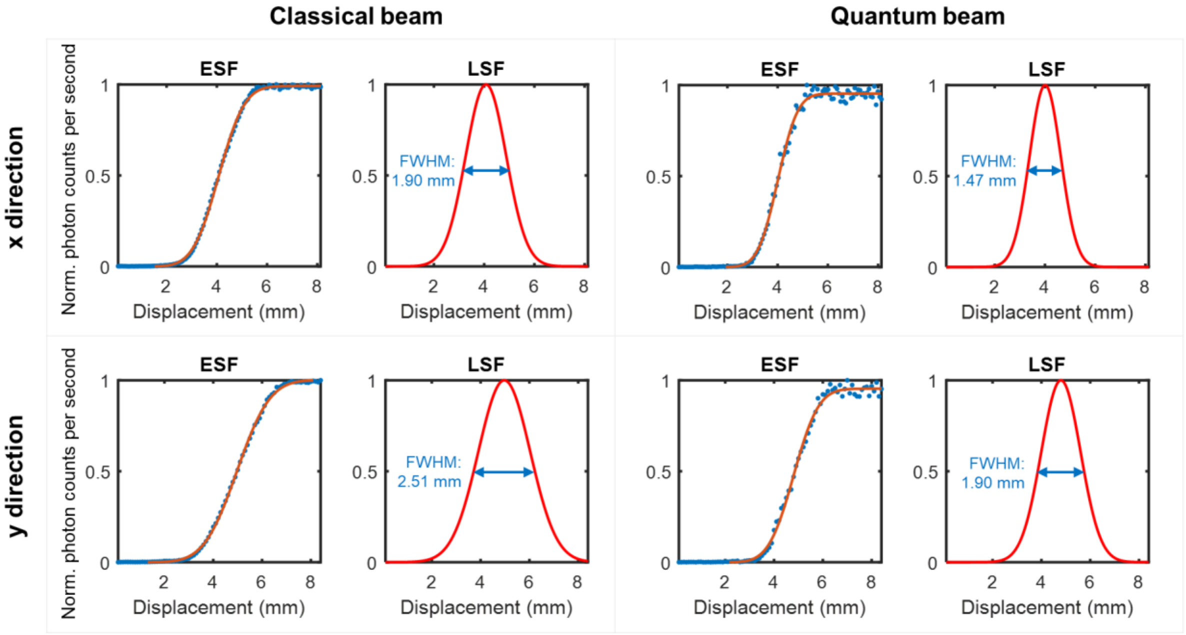

Fig. S7 Characterization of horizontal (x) and vertical (y) beam widths of classical imaging and ICE.

ESFs were acquired by scanning a sharp edge to block the beams. The FWHMs of the LSFs (derivatives of the ESFs) were used to estimate the beam widths. Norm., normalized.

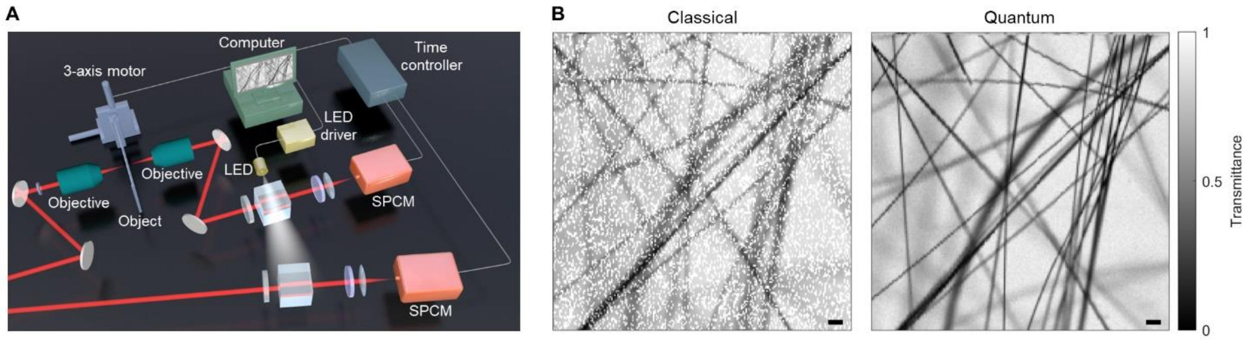

Fig. S8 ICE of carbon fibers in the presence of stray light.
(**A**) Experimental setup with the addition of a white light-emitting diode (LED) for randomly generated stray light. (**B**) Classical and ICE images of carbon fibers in the presence of stray light. Scale bars, 100 μm.

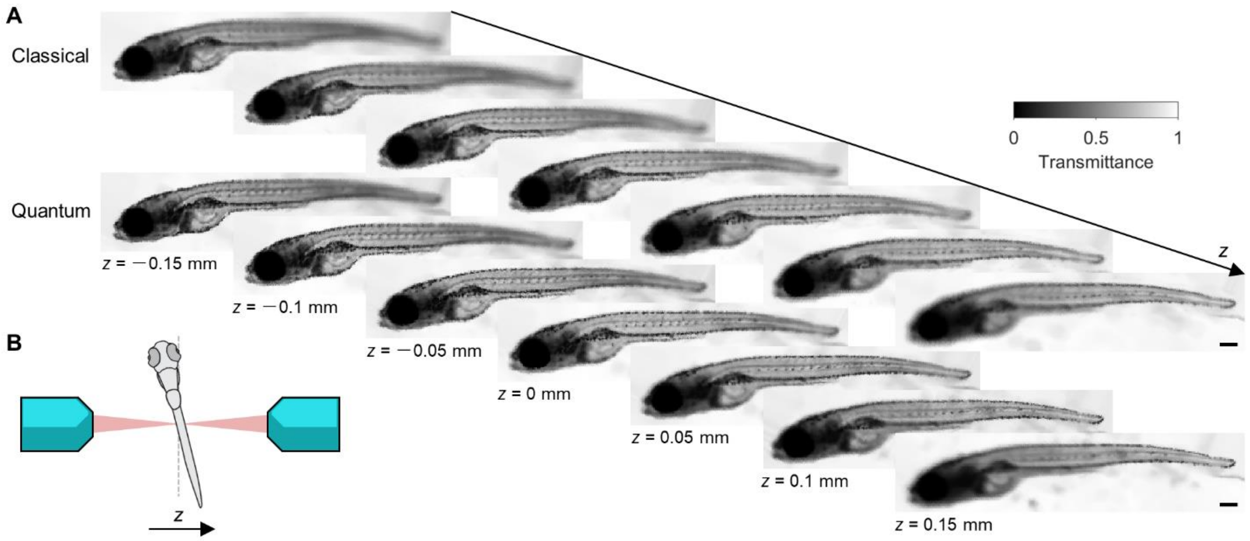

Fig. S9 ICE of a whole zebrafish.
(**A**) Classical and ICE images of an agarose-embedded zebrafish imaged at different *z* positions. Scale bars, 200 µm. (**B**) Illustration of the imaging configuration, where the torso of the zebrafish is oblique to the imaging plane.

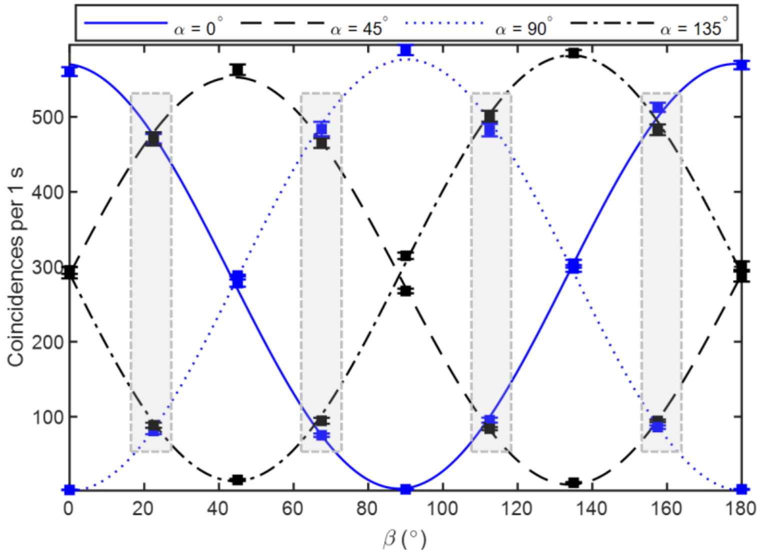

Fig. S10 Characterization of polarization entanglement through Bell's test.
Coincidence counts as a function of β acquired with 1 s integration time for $\alpha = 0°, 45°, 90°, 135°$. Experimental results are plotted as means ± standard errors of the means. The points marked by gray dashed rectangles are used to calculate the S_{CHSH} value. The curves are fits based on Eq. (S42).

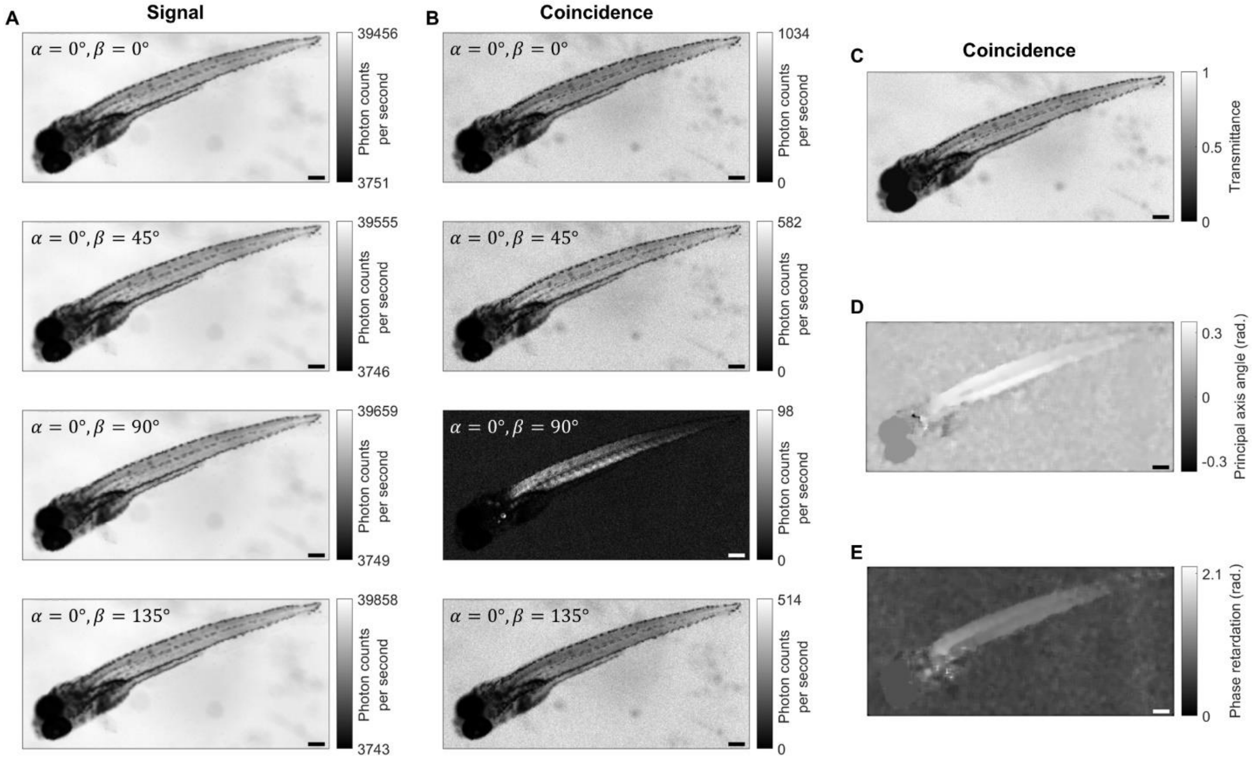

Fig. S11 Quantitative quantum birefringence imaging of a whole zebrafish using signal and coincidence counts.

(**A**) Classical images acquired with raw signal counts with constant α and variable β. (**B**) ICE images acquired with coincidence counts with constant α and variable β. (**C**–**E**) Transmittance (C), principal refractive index axis (D), and phase retardation between the two refractive index axes (E) calculated using the ICE images in (B). Scale bars, 200 μm.

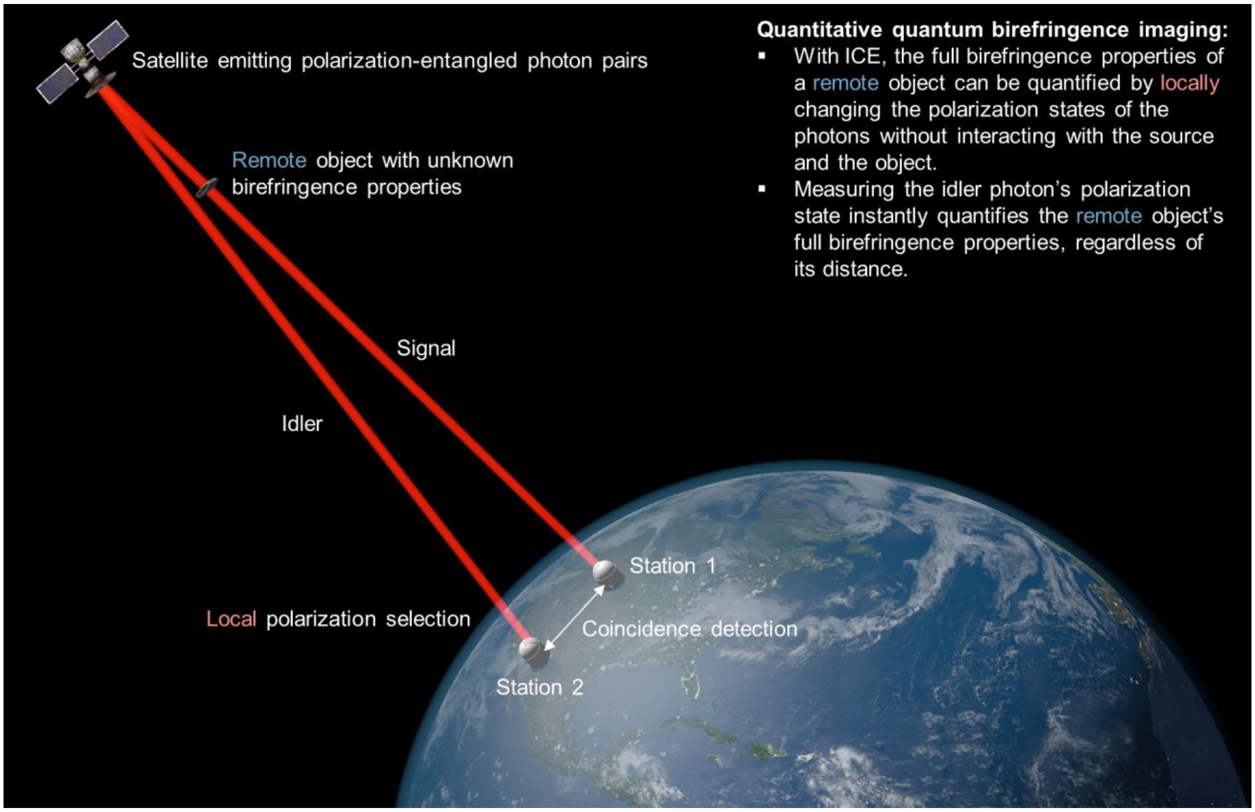

Fig. S12 Potential application of quantitatitve quantum birefringence imaging in remote sensing.

With a satellite emitting polarization-entangled photon pairs (*78*, *79*), ICE can quantify the birefringence properties of a remote object by changing the polarization states of the photons without interacting with the source and the object. Through polarization entanglement, measuring the idler photon's polarization state instantly determines the incident signal photon's and, consequently, the remote object's full birefringence properties, regardless of its distance.

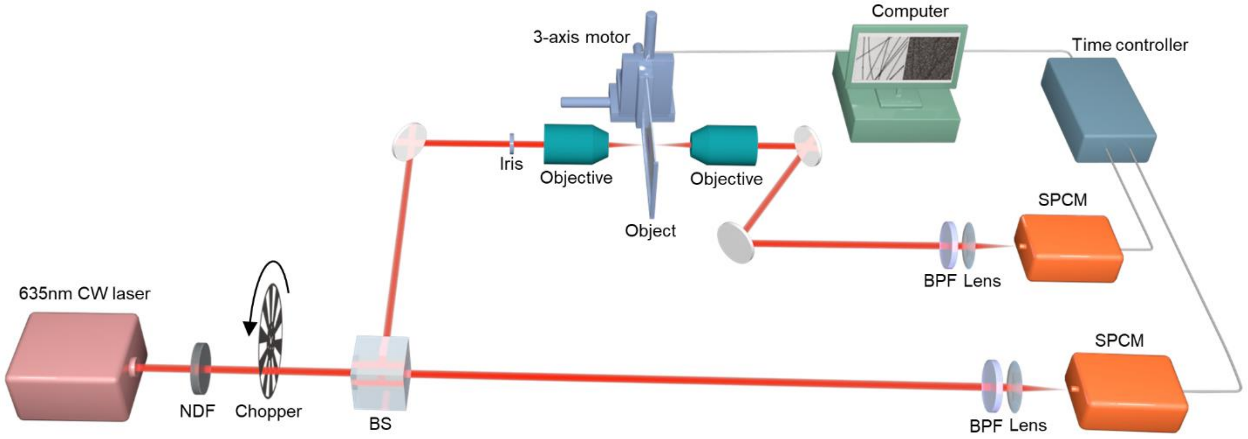

Fig. S13 Experimental setup of "ICE" with a classical source instead.
CW, continuous wave; NDF, neutral density filter; BS, beam splitter; BPF, band-pass filter; SPCM, single-photon counting module.

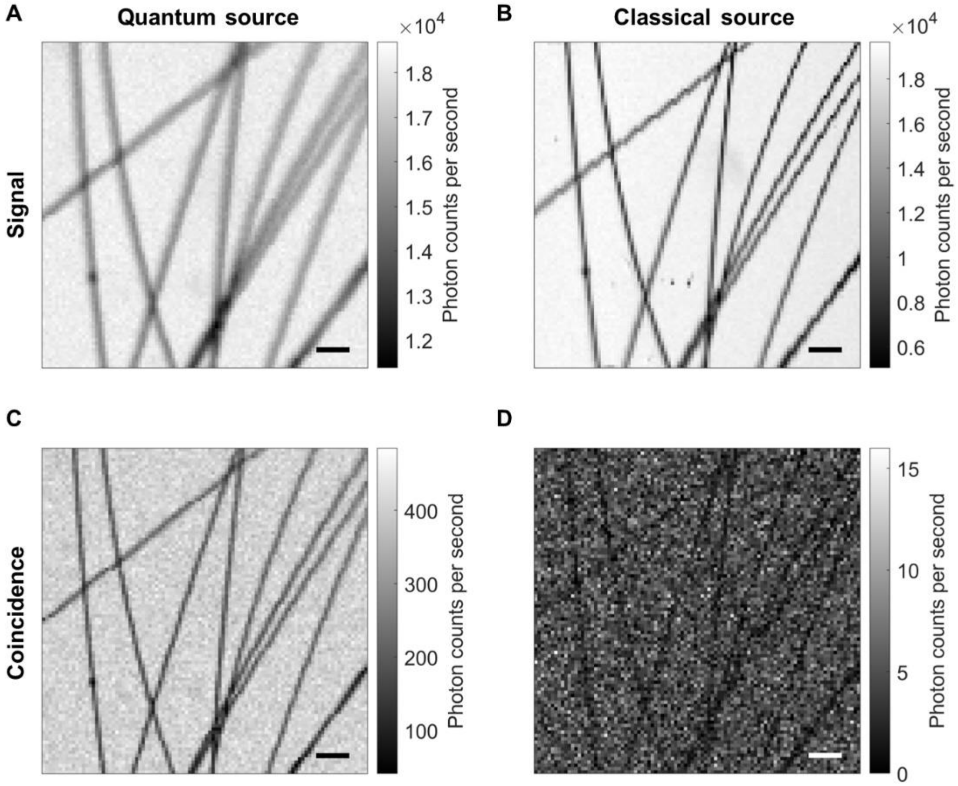

Fig. S14 ICE images of carbon fibers acquired using entangled and classical sources. (**A** and **B**) Images formed with the raw signal counts using the entangled (A) and classical (B) sources. (**C** and **D**) Images formed with the coincidence counts using the entangled (C) and classical (D) sources. Scale bars, 100 µm.

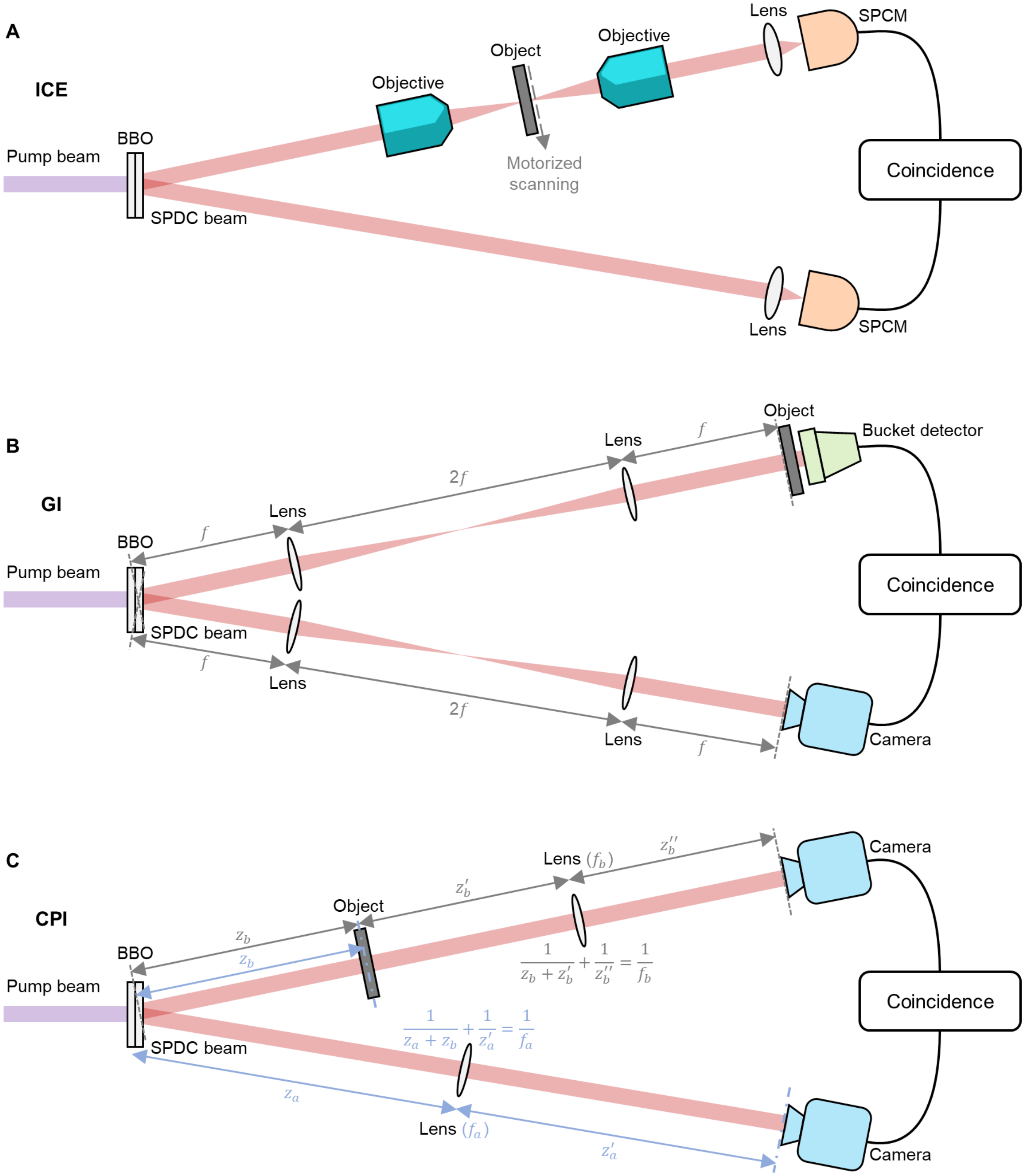

Fig. S15 Comparison of ICE, GI, and CPI.
(**A**) Schematic of ICE. The object is directly imaged to the SPCM in the signal arm. An image of the object is retrieved by recording the coincidence of the two SPCMs while raster scanning the object. (**B**) Schematic of GI. The BBO crystal is imaged to both the object and the camera. The bucket detector records all the photons transmitted through the object. Through coincidence detection, a ghost image of the object is retrieved from the camera and triggered by the bucket detector. (**C**) Schematic of CPI. The BBO crystal is imaged to the camera in the signal arm through the lens with a focal length of f_b, where the labeled distances satisfy the thin-lens equation

$1/(z_b + z_b') + 1/z_b'' = 1/f_b$. The ghost image of the object is imaged to the camera in the idler arm though the lens with a focal length of f_a, satisfying the condition $1/(z_a + z_b) + 1/z_b' = 1/f_a$. Through coincidence detection, a ghost image of the object is retrieved from the camera in the idler arm, triggered by the camera in the signal arm.

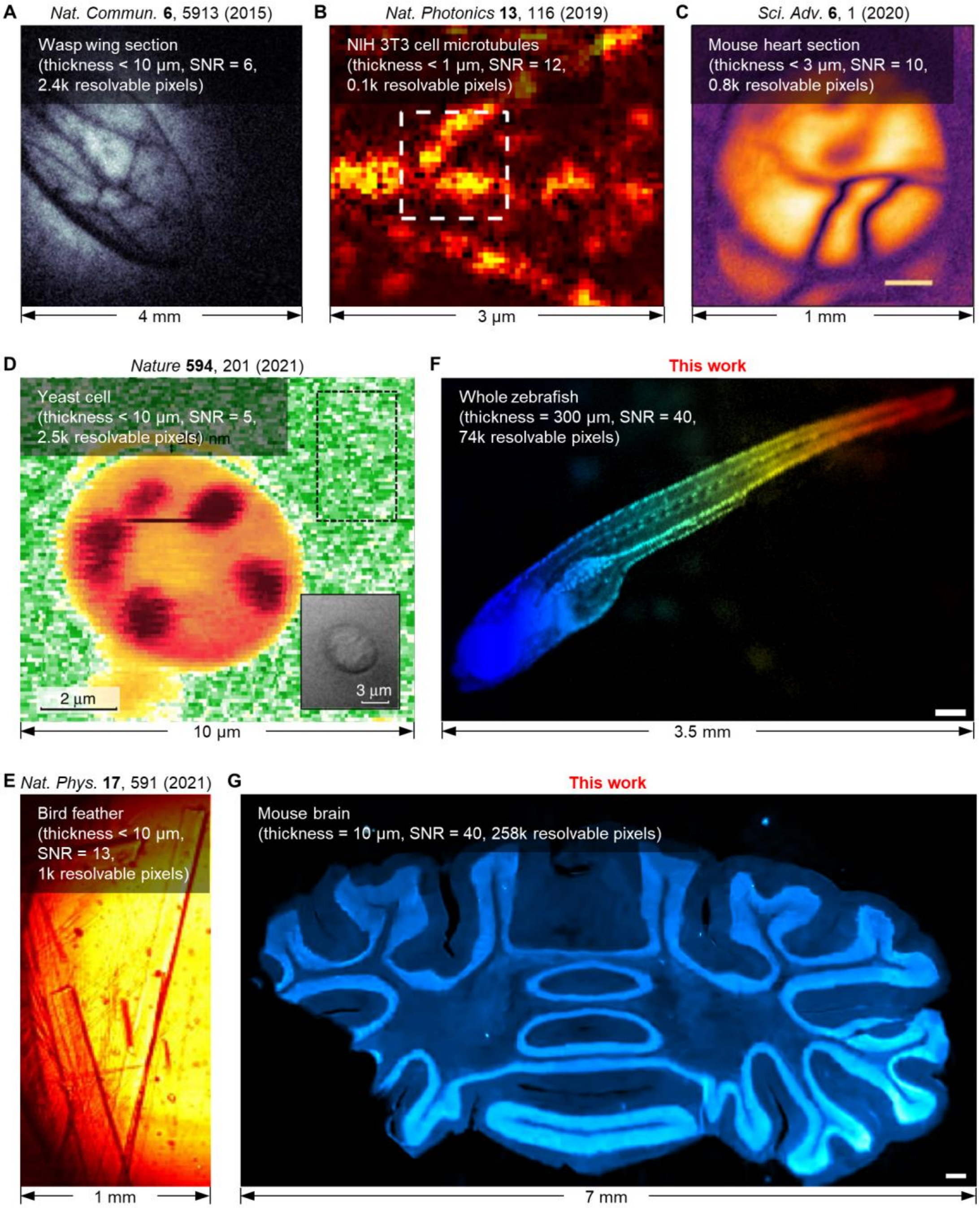

Fig. S16 Comparison of ICE and existing quantum bioimaging modalities.
(**A**) Quantum image of a wasp wing in a 4 mm × 4 mm FOV [adapted with permission from Morris *et al.* (*7*)]. (**B**) Quantum image of a 3 μm × 3 μm section of microtubules in a fixed 3T3 cell labeled with fluorescent quantum dots [adapted with permission from Tenne *et al.* (*8*)]. (**C**) Quantum image of the histology sample of a mouse heart in a 1 mm × 1 mm FOV [adapted with permission from Kviatkovsky *et al.* (*9*)]. (**D**) Quantum image of a yeast cell in aqueous buffer in a 10 μm × 10 μm FOV [adapted with permission from Casacio *et al.* (*11*)]. (**E**) Quantum image of parts of a bird feather in a 2 mm × 1 mm FOV [adapted with permission from Defienne *et al.* (*10*)]. (**F** and **G**) Quantum images of a whole zebrafish in a 3.5 mm × 2.3 mm FOV (F) and a mouse brain slice in a 7 mm × 4 mm FOV (G), presented in this work.

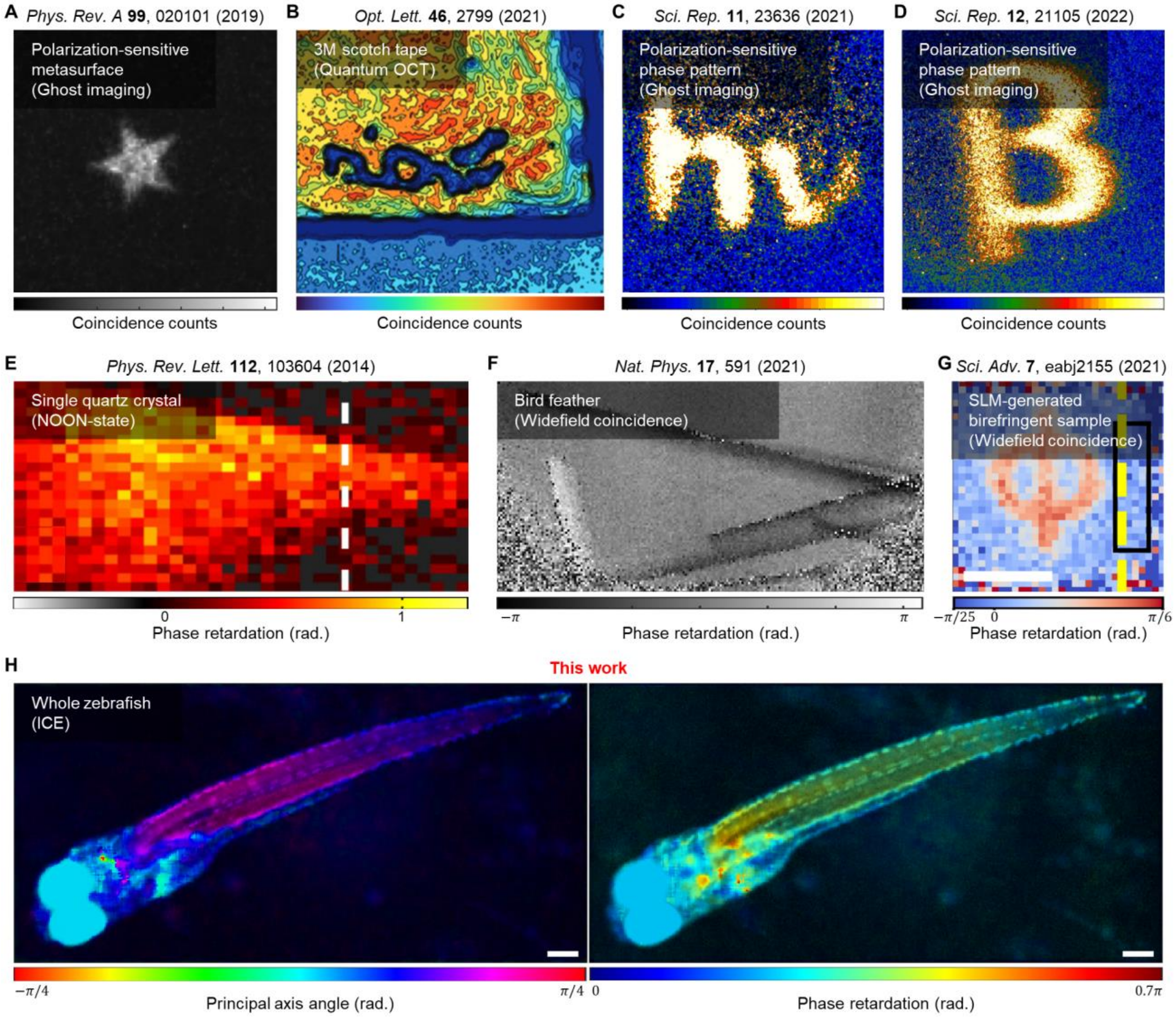

Fig. S17 Comparison of ICE and existing quantum birefringence imaging techniques. (**A**) Quantum ghost image of a polarization-sensitive metasurface [adapted with permission from Altuzarra *et al.* (*38*)]. (**B**) Polarization-sensitive quantum optical coherence tomography (OCT) image of a birefringent 3M scotch tape [adapted with permission from Sukharenko *et al.* (*39*)]. (**C**) Quantum ghost image of a polarization-sensitive phase pattern [adapted with permission from Kaur *et al.* (*40*)]. (**D**) Quantum ghost image of a polarization-sensitive phase pattern [adapted with permission from Saxena *et al.* (*41*)]. (**E**) Quantum polarization microscopy phase image of a sample of single quartz crystal using $N = 2$ NOON states [adapted with permission from Israel *et al.* (*42*)]. (**F**) Quantum holographic phase image of parts of a bird feather [adapted with permission from Defienne *et al.* (*10*)]. (**G**) Quantum phase image of a birefringent test sample generated by a spatial light modulator (SLM) [adapted with permission from Camphausen *et al.* (*43*)]. (**H**) Quantum images of the principal refractive index axis angle (left) and the phase retardation between the two refractive index axes (right) of a whole zebrafish, presented in this work.

REFERENCES AND NOTES

1. Y. M. Sigal, R. Zhou, X. Zhuang, Visualizing and discovering cellular structures with super-resolution microscopy. *Science* **361**, 880–887 (2018).

2. L. Schermelleh, A. Ferrand, T. Huser, C. Eggeling, M. Sauer, O. Biehlmaier, G. P. C. Drummen, Super-resolution microscopy demystified. *Nat. Cell Biol.* **21**, 72–84 (2019).

3. N. Thekkek, R. Richards-Kortum, Optical imaging for cervical cancer detection: Solutions for a continuing global problem. *Nat. Rev. Cancer* **8**, 725–731 (2008).

4. T.-L. Liu, S. Upadhyayula, D. E. Milkie, V. Singh, K. Wang, I. A. Swinburne, K. R. Mosaliganti, Z. M. Collins, T. W. Hiscock, J. Shea, A. Q. Kohrman, T. N. Medwig, D. Dambournet, R. Forster, B. Cunniff, Y. Ruan, H. Yashiro, S. Scholpp, E. M. Meyerowitz, D. Hockemeyer, D. G. Drubin, B. L. Martin, D. Q. Matus, M. Koyama, S. G. Megason, T. Kirchhausen, E. Betzig, Observing the cell in its native state: Imaging subcellular dynamics in multicellular organisms. *Science* **360**, eaaq1392 (2018).

5. T. Zhang, O. Hernandez, R. Chrapkiewicz, A. Shai, M. J. Wagner, Y. Zhang, C.-H. Wu, J. Z. Li, M. Inoue, Y. Gong, B. Ahanonu, H. Zeng, H. Bito, M. J. Schnitzer, Kilohertz two-photon brain imaging in awake mice. *Nat. Methods* **16**, 1119–1122 (2019).

6. J. Wu, Y. Liang, S. Chen, C.-L. Hsu, M. Chavarha, S. W. Evans, D. Shi, M. Z. Lin, K. K. Tsia, N. Ji, Kilohertz two-photon fluorescence microscopy imaging of neural activity in vivo. *Nat. Methods* **17**, 287–290 (2020).

7. P. A. Morris, R. S. Aspden, J. E. C. Bell, R. W. Boyd, M. J. Padgett, Imaging with a small number of photons. *Nat. Commun.* **6**, 5913 (2015).

8. R. Tenne, U. Rossman, B. Rephael, Y. Israel, A. Krupinski-Ptaszek, R. Lapkiewicz, Y. Silberberg, D. Oron, Super-resolution enhancement by quantum image scanning microscopy. *Nat. Photonics* **13**, 116–122 (2019).

9. I. Kviatkovsky, H. M. Chrzanowski, E. G. Avery, H. Bartolomaeus, S. Ramelow, Microscopy with undetected photons in the mid-infrared. *Sci. Adv.* **6**, eabd0264 (2020).

10. H. Defienne, B. Ndagano, A. Lyons, D. Faccio, Polarization entanglement-enabled quantum holography. *Nat. Phys.* **17**, 591–597 (2021).

11. C. A. Casacio, L. S. Madsen, A. Terrasson, M. Waleed, K. Barnscheidt, B. Hage, M. A. Taylor, W. P. Bowen, Quantum-enhanced nonlinear microscopy. *Nature* **594**, 201–206 (2021).

12. F. Li, T. Li, M. O. Scully, G. S. Agarwal, Quantum advantage with seeded squeezed light for absorption measurement. *Phys. Rev. Appl.* **15**, 044030 (2021).

13. T. Li, F. Li, X. Liu, V. V. Yakovlev, G. S. Agarwal, Quantum-enhanced stimulated Brillouin scattering spectroscopy and imaging. *Optica* **9**, 959 (2022), 964.

14. M. Genovese, Real applications of quantum imaging. *J. Opt.* **18**, 073002 (2016).

15. P.-A. Moreau, E. Toninelli, T. Gregory, M. J. Padgett, Imaging with quantum states of light. *Nat. Rev. Phys.* **1**, 367–380 (2019).

16. G. Brida, M. Genovese, I. Ruo Berchera, Experimental realization of sub-shot-noise quantum imaging. *Nat. Photonics* **4**, 227–230 (2010).

17. M. A. Taylor, J. Janousek, V. Daria, J. Knittel, B. Hage, H.-A. Bachor, W. P. Bowen, Biological measurement beyond the quantum limit. *Nat. Photonics* **7**, 229–233 (2013).

18. P.-A. Moreau, J. Sabines-Chesterking, R. Whittaker, S. K. Joshi, P. M. Birchall, A. McMillan, J. G. Rarity, J. C. F. Matthews, Demonstrating an absolute quantum advantage in direct absorption measurement. *Sci. Rep.* **7**, 6256 (2017).

19. N. Samantaray, I. Ruo-Berchera, A. Meda, M. Genovese, Realization of the first sub-shot-noise wide field microscope. *Light Sci. Appl.* **6**, e17005 (2017).

20. I. R. Berchera, I. P. Degiovanni, Quantum imaging with sub-Poissonian light: Challenges and perspectives in optical metrology. *Metrologia* **56**, 024001 (2019).

21. J. Sabines-Chesterking, A. R. McMillan, P. A. Moreau, S. K. Joshi, S. Knauer, E. Johnston, J. G. Rarity, J. C. F. Matthews, Twin-beam sub-shot-noise raster-scanning microscope. *Opt. Express* **27**, 30810–30818 (2019).

22. E. Toninelli, M. P. Edgar, P.-A. Moreau, G. M. Gibson, G. D. Hammond, M. J. Padgett, Sub-shot-noise shadow sensing with quantum correlations. *Opt. Express* **25**, 21826–21840 (2017).

23. E. Knyazev, F. Ya. Khalili, M. V. Chekhova, Overcoming inefficient detection in sub-shot-noise absorption measurement and imaging. *Opt. Express* **27**, 7868–7885 (2019).

24. H. Defienne, M. Reichert, J. W. Fleischer, D. Faccio, Quantum image distillation. *Sci. Adv.* **5**, eaax0307 (2019).

25. T. Gregory, P.-A. Moreau, E. Toninelli, M. J. Padgett, Imaging through noise with quantum illumination. *Sci. Adv.* **6**, eaay2652 (2020).

26. O. Schwartz, J. M. Levitt, R. Tenne, S. Itzhakov, Z. Deutsch, D. Oron, Superresolution microscopy with quantum emitters. *Nano Lett.* **13**, 5832–5836 (2013).

27. J.-M. Cui, F.-W. Sun, X.-D. Chen, Z.-J. Gong, G.-C. Guo, Quantum statistical imaging of particles without restriction of the diffraction limit. *Phys. Rev. Lett.* **110**, 153901 (2013).

28. D. Gatto Monticone, K. Katamadze, P. Traina, E. Moreva, J. Forneris, I. Ruo-Berchera, P. Olivero, I. P. Degiovanni, G. Brida, M. Genovese, Beating the Abbe diffraction limit in confocal microscopy via nonclassical photon statistics. *Phys. Rev. Lett.* **113**, 143602 (2014).

29. Y. Israel, R. Tenne, D. Oron, Y. Silberberg, Quantum correlation enhanced super-resolution localization microscopy enabled by a fibre bundle camera. *Nat. Commun.* **8**, 14786 (2017).

30. M. Unternährer, B. Bessire, L. Gasparini, M. Perenzoni, A. Stefanov, Super-resolution quantum imaging at the Heisenberg limit. *Optica* **5**, 1150 (2018).

31. E. Toninelli, P.-A. Moreau, T. Gregory, A. Mihalyi, M. Edgar, N. Radwell, M. Padgett, Resolution-enhanced quantum imaging by centroid estimation of biphotons. *Optica* **6**, 347 (2019).

32. Z. He, Y. Zhang, X. Tong, L. Li, L. V. Wang, Quantum microscopy of cells at the Heisenberg limit. *Nat. Commun.* **14**, 2441 (2023).

33. M. A. Taylor, W. P. Bowen, Quantum metrology and its application in biology. arXiv:1409.0950 [quant-ph] (2016).

34. E. Losero, I. Ruo-Berchera, A. Meda, A. Avella, M. Genovese, Unbiased estimation of an optical loss at the ultimate quantum limit with twin-beams. *Sci. Rep.* **8**, 7431 (2018).

35. K. Morimoto, A. Ardelean, M.-L. Wu, A. C. Ulku, I. M. Antolovic, C. Bruschini, E. Charbon, Megapixel time-gated SPAD image sensor for 2D and 3D imaging applications. *Optica* **7**, 346 (2020).

36. K. Morimoto, J. Iwata, M. Shinohara, H. Sekine, A. Abdelghafar, H. Tsuchiya, Y. Kuroda, K. Tojima, W. Endo, Y. Maehashi, Y. Ota, T. Sasago, S. Maekawa, S. Hikosaka, T. Kanou, A. Kato, T. Tezuka, S. Yoshizaki, T. Ogawa, K. Uehira, A. Ehara, F. Inui, Y. Matsuno, K. Sakurai, T. Ichikawa, 3.2 megapixel 3D-stacked charge focusing SPAD for low-light imaging and depth sensing, in *2021 IEEE International Electron Devices Meeting (IEDM)* (IEEE, 2021).

37. J. Ma, D. Zhang, D. Robledo, L. Anzagira, S. Masoodian, Ultra-high-resolution quanta image sensor with reliable photon-number-resolving and high dynamic range capabilities. *Sci. Rep.* **12**, 13869 (2022).

38. C. Altuzarra, A. Lyons, G. Yuan, C. Simpson, T. Roger, J. S. Ben-Benjamin, D. Faccio, Imaging of polarization-sensitive metasurfaces with quantum entanglement. *Phys. Rev. A* **99**, 020101 (2019).

39. V. Sukharenko, S. Bikorimana, R. Dorsinville, Birefringence and scattering characterization using polarization sensitive quantum optical coherence tomography. *Opt. Lett.* **46**, 2799–2802 (2021).

40. M. Kaur, M. Singh, Quantum imaging of a polarisation sensitive phase pattern with hyper-entangled photons. *Sci. Rep.* **11**, 23636 (2021).

41. A. Saxena, M. Kaur, V. Devrari, M. Singh, Quantum ghost imaging of a transparent polarisation sensitive phase pattern. *Sci. Rep.* **12**, 21105 (2022).

42. Y. Israel, S. Rosen, Y. Silberberg, Supersensitive polarization microscopy using noon states of light. *Phys. Rev. Lett.* **112**, 103604 (2014).

43. R. Camphausen, Á. Cuevas, L. Duempelmann, R. A. Terborg, E. Wajs, S. Tisa, A. Ruggeri, I. Cusini, F. Steinlechner, V. Pruneri, A quantum-enhanced wide-field phase imager. *Sci. Adv.* **7**, eabj2155 (2021).

44. G. Grynberg, A. Aspect, C. Fabre, *Introduction to Quantum Optics: From the Semi-Classical Approach to Quantized Light* (Cambridge Univ. Press, 2012).

45. R. Daendliker, Concept of modes in optics and photonics, in *Sixth International Conference on Education and Training in Optics and Photonics* (SPIE, 2000), vol. 3831, pp. 193–198.

46. Z.-J. Cheng, Y. Yang, H.-Y. Huang, Q.-Y. Yue, C.-S. Guo, Single-shot quantitative birefringence microscopy for imaging birefringence parameters. *Opt. Lett.* **44**, 3018–3021 (2019).

47. Y. Yang, H.-Y. Huang, C.-S. Guo, Polarization holographic microscope slide for birefringence imaging of anisotropic samples in microfluidics. *Opt. Express* **28**–14773–14762 (2020).

48. C. Lane, D. Rode, T. Rösgen, Two-dimensional birefringence measurement technique using a polarization camera. *Appl. Optics* **60**, 8435–8444 (2021).

49. P. G. Kwiat, Hyper-entangled states. *J. Mod. Opt.* **44**, 2173–2184 (1997).

50. J. T. Barreiro, N. K. Langford, N. A. Peters, P. G. Kwiat, Generation of hyperentangled photon pairs. *Phys. Rev. Lett.* **95**, 260501 (2005).

51. J. Liu, Q. Yang, S. Chen, Z. Xiao, S. Wen, H. Luo, Intrinsic optical spatial differentiation enabled quantum dark-field microscopy. *Phys. Rev. Lett.* **128**, 193601 (2022).

52. B. Ndagano, H. Defienne, D. Branford, Y. D. Shah, A. Lyons, N. Westerberg, E. M. Gauger, D. Faccio, Quantum microscopy based on Hong–Ou–Mandel interference. *Nat. Photonics* **16**, 384–389 (2022).

53. H. Defienne, P. Cameron, B. Ndagano, A. Lyons, M. Reichert, J. Zhao, A. R. Harvey, E. Charbon, J. W. Fleischer, D. Faccio, Pixel super-resolution with spatially entangled photons. *Nat. Commun.* **13**, 3566 (2022).

54. B. Ndagano, H. Defienne, A. Lyons, I. Starshynov, F. Villa, S. Tisa, D. Faccio, Imaging and certifying high-dimensional entanglement with a single-photon avalanche diode camera. *Npj Quantum Inf.* **6**, 94 (2020).

55. Z. Wang, A. C. Bovik, H. R. Sheikh, E. P. Simoncelli, Image quality assessment: From error visibility to structural similarity. *IEEE Trans. Image Process.* **13**, 600–612 (2004).

56. P. G. Kwiat, E. Waks, A. G. White, I. Appelbaum, P. H. Eberhard, Ultrabright source of polarization-entangled photons. *Phys. Rev. A* **60**, R773–R776 (1999).

57. D. Dehlinger, M. W. Mitchell, Entangled photons, nonlocality, and Bell inequalities in the undergraduate laboratory. *Am. J. Phys.* **70**, 903–910 (2002).

58. J. F. Clauser, M. A. Horne, A. Shimony, R. A. Holt, Proposed experiment to test local hidden-variable theories. *Phys. Rev. Lett.* **23**, 880–884 (1969).

59. R. S. Bennink, S. J. Bentley, R. W. Boyd, "Two-photon" coincidence imaging with a classical source. *Phys. Rev. Lett.* **89**, 113601 (2002).

60. X.-H. Chen, Q. Liu, K.-H. Luo, L.-A. Wu, Lensless ghost imaging with true thermal light. *Opt. Lett.* **34**, 695–697 (2009).

61. O. Katz, Y. Bromberg, Y. Silberberg, Compressive ghost imaging. *Appl. Phys. Lett.* **95**, 131110 (2009).

62. P.-A. Moreau, E. Toninelli, T. Gregory, M. J. Padgett, Ghost imaging using optical correlations. *Laser Photonics Rev.* **12**, 1700143 (2018).

63. F. Di Lena, F. Pepe, A. Garuccio, M. D'Angelo, Correlation plenoptic imaging: An overview. *Appl. Sci.* **8**, 1958 (2018).

64. Y. Zhang, A. Orth, D. England, B. Sussman, Ray tracing with quantum correlated photons to image a three-dimensional scene. *Phys. Rev. A* **105**, L011701 (2022).

65. L. Li, Z. Liu, X. Ren, S. Wang, V.-C. Su, M.-K. Chen, C. H. Chu, H. Y. Kuo, B. Liu, W. Zang, G. Guo, L. Zhang, Z. Wang, S. Zhu, D. P. Tsai, Metalens-array–based high-dimensional and multiphoton quantum source. *Science* **368**, 1487–1490 (2020).

66. N. Tomm, A. Javadi, N. O. Antoniadis, D. Najer, M. C. Löbl, A. R. Korsch, R. Schott, S. R. Valentin, A. D. Wieck, A. Ludwig, R. J. Warburton, A bright and fast source of coherent single photons. *Nat. Nanotechnol.* **16**, 399–403 (2021).

67. A. Meda, E. Losero, N. Samantaray, F. Scafirimuto, S. Pradyumna, A. Avella, I. Ruo-Berchera, M. Genovese, Photon-number correlation for quantum enhanced imaging and sensing. *J. Opt.* **19**, 094002 (2017).

68. N. M. Phan, M. F. Cheng, D. A. Bessarab, L. A. Krivitsky, Interaction of fixed number of photons with retinal rod cells. *Phys. Rev. Lett.* **112**, 213601 (2014).

69. K. Dabov, A. Foi, V. Katkovnik, K. Egiazarian, Image denoising by sparse 3-D transform-domain collaborative filtering. *IEEE Trans. Image Process.* **16**, 2080–2095 (2007).

70. M. Makitalo, A. Foi, Optimal inversion of the generalized anscombe transformation for Poisson-Gaussian noise. *IEEE Trans. Image Process.* **22**, 91–103 (2013).

71. E. Jakeman, J. G. Rarity, The use of pair production processes to reduce quantum noise in transmission measurements. *Opt. Commun.* **59**, 219–223 (1986).

72. P. R. Tapster, S. F. Seward, J. G. Rarity, Sub-shot-noise measurement of modulated absorption using parametric down-conversion. *Phys. Rev. A* **44**, 3266–3269 (1991).

73. A. F. Abouraddy, B. E. A. Saleh, A. V Sergienko, M. C. Teich, Entangled-photon Fourier optics. *J. Opt. Soc. Am. B* **19**, 1174 (2002).

74. A. Anwar, P. Vaity, C. Perumangatt, R. P. Singh, Direct transfer of pump amplitude to parametric down-converted photons. *Opt. Lett.* **43**, 1155–1158 (2018).

75. S. B. Mehta, M. Shribak, R. Oldenbourg, Polarized light imaging of birefringence and diattenuation at high resolution and high sensitivity. *J. Opt.* **15**, 094007 (2013).

76. F. V. Pepe, F. Di Lena, A. Mazzilli, E. Edrei, A. Garuccio, G. Scarcelli, M. D’Angelo, Diffraction-limited plenoptic imaging with correlated light. *Phys. Rev. Lett.* **119**, 243602 (2017).

77. F. Pepe, F. Di Lena, A. Garuccio, G. Scarcelli, M. D’Angelo, Correlation plenoptic imaging with entangled photons. *Dent. Tech.* **4**, 17 (2016).

78. S.-K. Liao, W.-Q. Cai, W.-Y. Liu, L. Zhang, Y. Li, J.-G. Ren, J. Yin, Q. Shen, Y. Cao, Z.-P. Li, F.-Z. Li, X.-W. Chen, L.-H. Sun, J.-J. Jia, J.-C. Wu, X.-J. Jiang, J.-F. Wang, Y.-M. Huang, Q. Wang, Y.-L. Zhou, L. Deng, T. Xi, L. Ma, T. Hu, Q. Zhang, Y.-A. Chen, N.-L. Liu, X.-B. Wang, Z.-C. Zhu, C.-Y. Lu, R. Shu, C.-Z. Peng, J.-Y. Wang, J.-W. Pan, Satellite-to-ground quantum key distribution. *Nature* **549**, 43–47 (2017).

79. J. Yin, Y. Cao, Y.-H. Li, S.-K. Liao, L. Zhang, J.-G. Ren, W.-Q. Cai, W.-Y. Liu, B. Li, H. Dai, G.-B. Li, Q.-M. Lu, Y.-H. Gong, Y. Xu, S.-L. Li, F.-Z. Li, Y.-Y. Yin, Z.-Q. Jiang, M. Li, J.-J. Jia, G. Ren, D. He, Y.-L. Zhou, X.-X. Zhang, N. Wang, X. Chang, Z.-C. Zhu, N.-L. Liu, Y.-A. Chen, C.-Y. Lu, R. Shu, C.-Z. Peng, J.-Y. Wang, J.-W. Pan, Satellite-based entanglement distribution over 1200 kilometers. *Science* **356**, 1140–1144 (2017).

REFERENCES AND NOTES

1. Y. M. Sigal, R. Zhou, X. Zhuang, Visualizing and discovering cellular structures with super-resolution microscopy. *Science* **361**, 880–887 (2018).

2. L. Schermelleh, A. Ferrand, T. Huser, C. Eggeling, M. Sauer, O. Biehlmaier, G. P. C. Drummen, Super-resolution microscopy demystified. *Nat. Cell Biol.* **21**, 72–84 (2019).

3. N. Thekkek, R. Richards-Kortum, Optical imaging for cervical cancer detection: Solutions for a continuing global problem. *Nat. Rev. Cancer* **8**, 725–731 (2008).

4. T.-L. Liu, S. Upadhyayula, D. E. Milkie, V. Singh, K. Wang, I. A. Swinburne, K. R. Mosaliganti, Z. M. Collins, T. W. Hiscock, J. Shea, A. Q. Kohrman, T. N. Medwig, D. Dambournet, R. Forster, B. Cunniff, Y. Ruan, H. Yashiro, S. Scholpp, E. M. Meyerowitz, D. Hockemeyer, D. G. Drubin, B. L. Martin, D. Q. Matus, M. Koyama, S. G. Megason, T. Kirchhausen, E. Betzig, Observing the cell in its native state: Imaging subcellular dynamics in multicellular organisms. *Science* **360**, eaaq1392 (2018).

5. T. Zhang, O. Hernandez, R. Chrapkiewicz, A. Shai, M. J. Wagner, Y. Zhang, C.-H. Wu, J. Z. Li, M. Inoue, Y. Gong, B. Ahanonu, H. Zeng, H. Bito, M. J. Schnitzer, Kilohertz two-photon brain imaging in awake mice. *Nat. Methods* **16**, 1119–1122 (2019).

6. J. Wu, Y. Liang, S. Chen, C.-L. Hsu, M. Chavarha, S. W. Evans, D. Shi, M. Z. Lin, K. K. Tsia, N. Ji, Kilohertz two-photon fluorescence microscopy imaging of neural activity in vivo. *Nat. Methods* **17**, 287–290 (2020).

7. P. A. Morris, R. S. Aspden, J. E. C. Bell, R. W. Boyd, M. J. Padgett, Imaging with a small number of photons. *Nat. Commun.* **6**, 5913 (2015).

8. R. Tenne, U. Rossman, B. Rephael, Y. Israel, A. Krupinski-Ptaszek, R. Lapkiewicz, Y. Silberberg, D. Oron, Super-resolution enhancement by quantum image scanning microscopy. *Nat. Photonics* **13**, 116–122 (2019).

9. I. Kviatkovsky, H. M. Chrzanowski, E. G. Avery, H. Bartolomaeus, S. Ramelow, Microscopy with undetected photons in the mid-infrared. *Sci. Adv.* **6**, eabd0264 (2020).

10. H. Defienne, B. Ndagano, A. Lyons, D. Faccio, Polarization entanglement-enabled quantum holography. *Nat. Phys.* **17**, 591–597 (2021).

11. C. A. Casacio, L. S. Madsen, A. Terrasson, M. Waleed, K. Barnscheidt, B. Hage, M. A. Taylor, W. P. Bowen, Quantum-enhanced nonlinear microscopy. *Nature* **594**, 201–206 (2021).

12. F. Li, T. Li, M. O. Scully, G. S. Agarwal, Quantum advantage with seeded squeezed light for absorption measurement. *Phys. Rev. Appl.* **15**, 044030 (2021).

13. T. Li, F. Li, X. Liu, V. V. Yakovlev, G. S. Agarwal, Quantum-enhanced stimulated Brillouin scattering spectroscopy and imaging. *Optica* **9**, 959 (2022), 964.

14. M. Genovese, Real applications of quantum imaging. *J. Opt.* **18**, 073002 (2016).

15. P.-A. Moreau, E. Toninelli, T. Gregory, M. J. Padgett, Imaging with quantum states of light. *Nat. Rev. Phys.* **1**, 367–380 (2019).

16. G. Brida, M. Genovese, I. Ruo Berchera, Experimental realization of sub-shot-noise quantum imaging. *Nat. Photonics* **4**, 227–230 (2010).

17. M. A. Taylor, J. Janousek, V. Daria, J. Knittel, B. Hage, H.-A. Bachor, W. P. Bowen, Biological measurement beyond the quantum limit. *Nat. Photonics* **7**, 229–233 (2013).

18. P.-A. Moreau, J. Sabines-Chesterking, R. Whittaker, S. K. Joshi, P. M. Birchall, A. McMillan, J. G. Rarity, J. C. F. Matthews, Demonstrating an absolute quantum advantage in direct absorption measurement. *Sci. Rep.* **7**, 6256 (2017).

19. N. Samantaray, I. Ruo-Berchera, A. Meda, M. Genovese, Realization of the first sub-shot-noise wide field microscope. *Light Sci. Appl.* **6**, e17005 (2017).

20. I. R. Berchera, I. P. Degiovanni, Quantum imaging with sub-Poissonian light: Challenges and perspectives in optical metrology. *Metrologia* **56**, 024001 (2019).

21. J. Sabines-Chesterking, A. R. McMillan, P. A. Moreau, S. K. Joshi, S. Knauer, E. Johnston, J. G. Rarity, J. C. F. Matthews, Twin-beam sub-shot-noise raster-scanning microscope. *Opt. Express* **27**, 30810–30818 (2019).

22. E. Toninelli, M. P. Edgar, P.-A. Moreau, G. M. Gibson, G. D. Hammond, M. J. Padgett, Sub-shot-noise shadow sensing with quantum correlations. *Opt. Express* **25**, 21826–21840 (2017).

23. E. Knyazev, F. Ya. Khalili, M. V. Chekhova, Overcoming inefficient detection in sub-shot-noise absorption measurement and imaging. *Opt. Express* **27**, 7868–7885 (2019).

24. H. Defienne, M. Reichert, J. W. Fleischer, D. Faccio, Quantum image distillation. *Sci. Adv.* **5**, eaax0307 (2019).

25. T. Gregory, P.-A. Moreau, E. Toninelli, M. J. Padgett, Imaging through noise with quantum illumination. *Sci. Adv.* **6**, eaay2652 (2020).

26. O. Schwartz, J. M. Levitt, R. Tenne, S. Itzhakov, Z. Deutsch, D. Oron, Superresolution microscopy with quantum emitters. *Nano Lett.* **13**, 5832–5836 (2013).

27. J.-M. Cui, F.-W. Sun, X.-D. Chen, Z.-J. Gong, G.-C. Guo, Quantum statistical imaging of particles without restriction of the diffraction limit. *Phys. Rev. Lett.* **110**, 153901 (2013).

28. D. Gatto Monticone, K. Katamadze, P. Traina, E. Moreva, J. Forneris, I. Ruo-Berchera, P. Olivero, I. P. Degiovanni, G. Brida, M. Genovese, Beating the Abbe diffraction limit in confocal microscopy via nonclassical photon statistics. *Phys. Rev. Lett.* **113**, 143602 (2014).

29. Y. Israel, R. Tenne, D. Oron, Y. Silberberg, Quantum correlation enhanced super-resolution localization microscopy enabled by a fibre bundle camera. *Nat. Commun.* **8**, 14786 (2017).

30. M. Unternährer, B. Bessire, L. Gasparini, M. Perenzoni, A. Stefanov, Super-resolution quantum imaging at the Heisenberg limit. *Optica* **5**, 1150 (2018).

31. E. Toninelli, P.-A. Moreau, T. Gregory, A. Mihalyi, M. Edgar, N. Radwell, M. Padgett, Resolution-enhanced quantum imaging by centroid estimation of biphotons. *Optica* **6**, 347 (2019).

32. Z. He, Y. Zhang, X. Tong, L. Li, L. V. Wang, Quantum microscopy of cells at the Heisenberg limit. *Nat. Commun.* **14**, 2441 (2023).

33. M. A. Taylor, W. P. Bowen, Quantum metrology and its application in biology. arXiv:1409.0950 [quant-ph] (2016).

34. E. Losero, I. Ruo-Berchera, A. Meda, A. Avella, M. Genovese, Unbiased estimation of an optical loss at the ultimate quantum limit with twin-beams. *Sci. Rep.* **8**, 7431 (2018).

35. K. Morimoto, A. Ardelean, M.-L. Wu, A. C. Ulku, I. M. Antolovic, C. Bruschini, E. Charbon, Megapixel time-gated SPAD image sensor for 2D and 3D imaging applications. *Optica* **7**, 346 (2020).

36. K. Morimoto, J. Iwata, M. Shinohara, H. Sekine, A. Abdelghafar, H. Tsuchiya, Y. Kuroda, K. Tojima, W. Endo, Y. Maehashi, Y. Ota, T. Sasago, S. Maekawa, S. Hikosaka, T. Kanou, A. Kato, T. Tezuka, S. Yoshizaki, T. Ogawa, K. Uehira, A. Ehara, F. Inui, Y. Matsuno, K. Sakurai, T. Ichikawa, 3.2 megapixel 3D-stacked charge focusing SPAD for low-light imaging and depth sensing, in *2021 IEEE International Electron Devices Meeting (IEDM)* (IEEE, 2021).

37. J. Ma, D. Zhang, D. Robledo, L. Anzagira, S. Masoodian, Ultra-high-resolution quanta image sensor with reliable photon-number-resolving and high dynamic range capabilities. *Sci. Rep.* **12**, 13869 (2022).

38. C. Altuzarra, A. Lyons, G. Yuan, C. Simpson, T. Roger, J. S. Ben-Benjamin, D. Faccio, Imaging of polarization-sensitive metasurfaces with quantum entanglement. *Phys. Rev. A* **99**, 020101 (2019).

39. V. Sukharenko, S. Bikorimana, R. Dorsinville, Birefringence and scattering characterization using polarization sensitive quantum optical coherence tomography. *Opt. Lett.* **46**, 2799–2802 (2021).

40. M. Kaur, M. Singh, Quantum imaging of a polarisation sensitive phase pattern with hyper-entangled photons. *Sci. Rep.* **11**, 23636 (2021).

41. A. Saxena, M. Kaur, V. Devrari, M. Singh, Quantum ghost imaging of a transparent polarisation sensitive phase pattern. *Sci. Rep.* **12**, 21105 (2022).

42. Y. Israel, S. Rosen, Y. Silberberg, Supersensitive polarization microscopy using noon states of light. *Phys. Rev. Lett.* **112**, 103604 (2014).

43. R. Camphausen, Á. Cuevas, L. Duempelmann, R. A. Terborg, E. Wajs, S. Tisa, A. Ruggeri, I. Cusini, F. Steinlechner, V. Pruneri, A quantum-enhanced wide-field phase imager. *Sci. Adv.* **7**, eabj2155 (2021).

44. G. Grynberg, A. Aspect, C. Fabre, *Introduction to Quantum Optics: From the Semi-Classical Approach to Quantized Light* (Cambridge Univ. Press, 2012).

45. R. Daendliker, Concept of modes in optics and photonics, in *Sixth International Conference on Education and Training in Optics and Photonics* (SPIE, 2000), vol. 3831, pp. 193–198.

46. Z.-J. Cheng, Y. Yang, H.-Y. Huang, Q.-Y. Yue, C.-S. Guo, Single-shot quantitative birefringence microscopy for imaging birefringence parameters. *Opt. Lett.* **44**, 3018–3021 (2019).

47. Y. Yang, H.-Y. Huang, C.-S. Guo, Polarization holographic microscope slide for birefringence imaging of anisotropic samples in microfluidics. *Opt. Express* **28**–14773–14762 (2020).

48. C. Lane, D. Rode, T. Rösgen, Two-dimensional birefringence measurement technique using a polarization camera. *Appl. Optics* **60**, 8435–8444 (2021).

49. P. G. Kwiat, Hyper-entangled states. *J. Mod. Opt.* **44**, 2173–2184 (1997).

50. J. T. Barreiro, N. K. Langford, N. A. Peters, P. G. Kwiat, Generation of hyperentangled photon pairs. *Phys. Rev. Lett.* **95**, 260501 (2005).

51. J. Liu, Q. Yang, S. Chen, Z. Xiao, S. Wen, H. Luo, Intrinsic optical spatial differentiation enabled quantum dark-field microscopy. *Phys. Rev. Lett.* **128**, 193601 (2022).

52. B. Ndagano, H. Defienne, D. Branford, Y. D. Shah, A. Lyons, N. Westerberg, E. M. Gauger, D. Faccio, Quantum microscopy based on Hong–Ou–Mandel interference. *Nat. Photonics* **16**, 384–389 (2022).

53. H. Defienne, P. Cameron, B. Ndagano, A. Lyons, M. Reichert, J. Zhao, A. R. Harvey, E. Charbon, J. W. Fleischer, D. Faccio, Pixel super-resolution with spatially entangled photons. *Nat. Commun.* **13**, 3566 (2022).

54. B. Ndagano, H. Defienne, A. Lyons, I. Starshynov, F. Villa, S. Tisa, D. Faccio, Imaging and certifying high-dimensional entanglement with a single-photon avalanche diode camera. *Npj Quantum Inf.* **6**, 94 (2020).

55. Z. Wang, A. C. Bovik, H. R. Sheikh, E. P. Simoncelli, Image quality assessment: From error visibility to structural similarity. *IEEE Trans. Image Process.* **13**, 600–612 (2004).

56. P. G. Kwiat, E. Waks, A. G. White, I. Appelbaum, P. H. Eberhard, Ultrabright source of polarization-entangled photons. *Phys. Rev. A* **60**, R773–R776 (1999).

57. D. Dehlinger, M. W. Mitchell, Entangled photons, nonlocality, and Bell inequalities in the undergraduate laboratory. *Am. J. Phys.* **70**, 903–910 (2002).

58. J. F. Clauser, M. A. Horne, A. Shimony, R. A. Holt, Proposed experiment to test local hidden-variable theories. *Phys. Rev. Lett.* **23**, 880–884 (1969).

59. R. S. Bennink, S. J. Bentley, R. W. Boyd, "Two-photon" coincidence imaging with a classical source. *Phys. Rev. Lett.* **89**, 113601 (2002).

60. X.-H. Chen, Q. Liu, K.-H. Luo, L.-A. Wu, Lensless ghost imaging with true thermal light. *Opt. Lett.* **34**, 695–697 (2009).

61. O. Katz, Y. Bromberg, Y. Silberberg, Compressive ghost imaging. *Appl. Phys. Lett.* **95**, 131110 (2009).

62. P.-A. Moreau, E. Toninelli, T. Gregory, M. J. Padgett, Ghost imaging using optical correlations. *Laser Photonics Rev.* **12**, 1700143 (2018).

63. F. Di Lena, F. Pepe, A. Garuccio, M. D'Angelo, Correlation plenoptic imaging: An overview. *Appl. Sci.* **8**, 1958 (2018).

64. Y. Zhang, A. Orth, D. England, B. Sussman, Ray tracing with quantum correlated photons to image a three-dimensional scene. *Phys. Rev. A* **105**, L011701 (2022).

65. L. Li, Z. Liu, X. Ren, S. Wang, V.-C. Su, M.-K. Chen, C. H. Chu, H. Y. Kuo, B. Liu, W. Zang, G. Guo, L. Zhang, Z. Wang, S. Zhu, D. P. Tsai, Metalens-array–based high-dimensional and multiphoton quantum source. *Science* **368**, 1487–1490 (2020).

66. N. Tomm, A. Javadi, N. O. Antoniadis, D. Najer, M. C. Löbl, A. R. Korsch, R. Schott, S. R. Valentin, A. D. Wieck, A. Ludwig, R. J. Warburton, A bright and fast source of coherent single photons. *Nat. Nanotechnol.* **16**, 399–403 (2021).

67. A. Meda, E. Losero, N. Samantaray, F. Scafirimuto, S. Pradyumna, A. Avella, I. Ruo-Berchera, M. Genovese, Photon-number correlation for quantum enhanced imaging and sensing. *J. Opt.* **19**, 094002 (2017).

68. N. M. Phan, M. F. Cheng, D. A. Bessarab, L. A. Krivitsky, Interaction of fixed number of photons with retinal rod cells. *Phys. Rev. Lett.* **112**, 213601 (2014).

69. K. Dabov, A. Foi, V. Katkovnik, K. Egiazarian, Image denoising by sparse 3-D transform-domain collaborative filtering. *IEEE Trans. Image Process.* **16**, 2080–2095 (2007).

70. M. Makitalo, A. Foi, Optimal inversion of the generalized anscombe transformation for Poisson-Gaussian noise. *IEEE Trans. Image Process.* **22**, 91–103 (2013).

71. E. Jakeman, J. G. Rarity, The use of pair production processes to reduce quantum noise in transmission measurements. *Opt. Commun.* **59**, 219–223 (1986).

72. P. R. Tapster, S. F. Seward, J. G. Rarity, Sub-shot-noise measurement of modulated absorption using parametric down-conversion. *Phys. Rev. A* **44**, 3266–3269 (1991).

73. A. F. Abouraddy, B. E. A. Saleh, A. V Sergienko, M. C. Teich, Entangled-photon Fourier optics. *J. Opt. Soc. Am. B* **19**, 1174 (2002).

74. A. Anwar, P. Vaity, C. Perumangatt, R. P. Singh, Direct transfer of pump amplitude to parametric down-converted photons. *Opt. Lett.* **43**, 1155–1158 (2018).

75. S. B. Mehta, M. Shribak, R. Oldenbourg, Polarized light imaging of birefringence and diattenuation at high resolution and high sensitivity. *J. Opt.* **15**, 094007 (2013).

76. F. V. Pepe, F. Di Lena, A. Mazzilli, E. Edrei, A. Garuccio, G. Scarcelli, M. D'Angelo, Diffraction-limited plenoptic imaging with correlated light. *Phys. Rev. Lett.* **119**, 243602 (2017).

77. F. Pepe, F. Di Lena, A. Garuccio, G. Scarcelli, M. D'Angelo, Correlation plenoptic imaging with entangled photons. *Dent. Tech.* **4**, 17 (2016).

78. S.-K. Liao, W.-Q. Cai, W.-Y. Liu, L. Zhang, Y. Li, J.-G. Ren, J. Yin, Q. Shen, Y. Cao, Z.-P. Li, F.-Z. Li, X.-W. Chen, L.-H. Sun, J.-J. Jia, J.-C. Wu, X.-J. Jiang, J.-F. Wang, Y.-M. Huang, Q. Wang, Y.-L. Zhou, L. Deng, T. Xi, L. Ma, T. Hu, Q. Zhang, Y.-A. Chen, N.-L. Liu, X.-B. Wang, Z.-C. Zhu, C.-Y. Lu, R. Shu, C.-Z. Peng, J.-Y. Wang, J.-W. Pan, Satellite-to-ground quantum key distribution. *Nature* **549**, 43–47 (2017).

79. J. Yin, Y. Cao, Y.-H. Li, S.-K. Liao, L. Zhang, J.-G. Ren, W.-Q. Cai, W.-Y. Liu, B. Li, H. Dai, G.-B. Li, Q.-M. Lu, Y.-H. Gong, Y. Xu, S.-L. Li, F.-Z. Li, Y.-Y. Yin, Z.-Q. Jiang, M. Li, J.-J. Jia, G. Ren, D. He, Y.-L. Zhou, X.-X. Zhang, N. Wang, X. Chang, Z.-C. Zhu, N.-L. Liu, Y.-A. Chen, C.-Y. Lu, R. Shu, C.-Z. Peng, J.-Y. Wang, J.-W. Pan, Satellite-based entanglement distribution over 1200 kilometers. *Science* **356**, 1140–1144 (2017).
